# Are long-term care systems aligned with person-centered integrated care? Evidence from the Western Pacific

**DOI:** 10.1016/j.healthpol.2025.105442

**Published:** 2025-12

**Authors:** Dongkyu Lee, Soojung Kim, Soonman Kwon, Sunghun Yun, Mikiko Kanda, Siwon Lee, Nicole Sutton, Paul Ong, Andy Inder

**Affiliations:** a Institute of Health and Environment, Seoul National University, Seoul, South Korea; b Graduate School of Public Health, Seoul National University, Seoul, South Korea; c Healthy Ageing unit, Division of Healthy Environments and Populations, World Health Organization Regional Office for the Western Pacific, Manila, Philippines; d Accounting Discipline Group, University of Technology Sydney, Sydney, Australia; e Tsao foundation, Singapore; f Ageing well, Health New Zealand, Wellington, New Zealand

**Keywords:** Person-centered care, Integrated care, Long-term care, Ageing in place, Western Pacific

## Abstract

**Background:**

Many Western Pacific countries have established long-term care (LTC) systems to support their rapidly aging populations. However, the extent to which these systems align with integrated care principles that enable individuals to age in place (AIP) remains unclear. Effective integration of LTC with healthcare is essential to enhance continuity of care, improve outcomes, and support AIP.

**Objective:**

This study examines the alignment of LTC policies in five Western Pacific countries—Australia, Japan, New Zealand, Republic of Korea, and Singapore—with integrated LTC principles. The analysis identifies systemic enablers and challenges in governance, financing, workforce, service delivery, information, monitoring & evaluation (IM&E), and innovation & research.

**Methods:**

Using an adapted World Health Organization LTC framework, we conducted a comparative analysis of the selected countries’ LTC policies.

**Results:**

All five countries emphasize aging in place and provide both institutional and community-based LTC services. However, key enablers of integration are often lacking. Fragmentation between LTC and healthcare is common, and coordination mechanisms such as care planning are hindered by inadequate accountability mechanisms due to misaligned incentives, challenges in funding integration, and often underdeveloped information systems for monitoring integrated care.

**Conclusion:**

Western Pacific LTC systems are not yet fully aligned with effective integrated LTC. Strengthening coordinated and accountable governance, integrating financing streams and incentive, enhancing IM&E systems for performance management, and leveraging innovation are crucial to enhancing integrated LTC in the region.

## Highlights

• Western Pacific LTC systems are not fully aligned with effective integrated LTC.
• Key system enablers fostering accountability for integrated care remain limited.
• Policy reforms should prioritize shared accountability, aligned financing, and data systems.

## 1 Background

More than one-third of the world’s population aged 65 and over resides in the Western Pacific Region (WPR), and the number is expected to almost double by 2040 [1]. While factors such as increased life expectancy, women’s education attainment and labor participation, and urbanization could be generally considered fruits of development, they also increase the mismatch between care needs and the supply of long-term care (LTC). Specifically, longer lifespans contribute to a greater number of older individuals potentially requiring care over extended periods, while societal changes such as increased female labor participation and evolving family structures linked to urbanization often reduce the availability of traditional informal caregivers. To deal with this mismatch, many countries have pursued the development of a public LTC system [2]. Several reasons for public engagement exist. First, the market for private LTC insurance is hard to grow on its own due to various factors including information asymmetry [3,4]. Second, the family caregiving burden could hinder labor participation and working hours, especially among women [5,6]. Third, lack of caregiving could result in more costly social admissions or hospital bed-blocking, and therefore, introducing a public LTC system could be a more cost-efficient option [7,8]. Fourth, lack of formalized caregiving could result in abuse and deterioration of well-being for both caretakers and caregivers [9].

LTC refers to services that support individuals with long-term physical or mental limitations, helping them maintain independent living despite difficulties in performing daily activities [10]. A primary objective of LTC policy is often to support Ageing in Place (AIP), allowing older adults to remain in their preferred community environments where they can sustain social activities and lead healthier lives [11]. This goal aligns with older adults' preferences and is generally considered more cost-effective than institutional care [12], leading many countries to shift from facility-centered models towards those supporting AIP [2].

Person-centered integrated LTC is another important principle supported by the literature, based on the notion that while the individual in need of multiple services could be considered as a boundary object [13], providers are often fragmented, resulting in either unmet needs or inefficiency and creates needs for collaboration [13,14]. The major goal of integrated care is to ensure continuity of care, fulfill unmet needs, and improve efficiency [11]. The key idea of such an approach might be that there must be a system enhancing coordination and collaboration across the health and social care sectors and responding to the needs of an individual, linked to the importance of needs assessment-based resource coordination mechanism putting person at the center [14]. However, while evidence showing the effectiveness of integrated LTC service models is common in the literature [15], study results on the effects of integrated care as a whole are inconsistent [16].

Due to the variability of the surrounding governance structure and its inherent complexity, it could be argued that an integrated care model, which had proven effectiveness in one environment, may not work in the other. This suggests that integrated care should be understood as a complex strategy to innovate and implement change in the way health and social care services are being delivered which involves multiple layers of the system [16,17]. The system-level enablers, or contexts, could be roughly understood as the broader institutional-level governance structure that pushes the fragmented providers to cooperate to achieve shared visions: improving health, reducing costs, and enhancing patient experience - often referred to as the triple aim of the healthcare system [18,19]. The literature suggests that developing a system that enables the delivery of integrated LTC requires well-functioning governance that effectively assigns accountability to the stakeholders. This includes the functional integration of financing and legislation, as well as the normative integration of values and visions between the stakeholders [20,21].

Although several studies suggesting theoretical frameworks for integrated LTC systems have been published [10,[20], [21], [22], [23]], actual assessments of country-level systems have rarely been reported from such perspectives. To the best of our knowledge, studies analyzing LTC systems of WPR countries have focused on the typology [[24], [25], [26]], reforms [2], financing system [[27], [28], [29]], cost-containment policies [30], fund allocation and equity [31], and developmental trajectories [32], most being conducted as a part of OECD countries. Most analyses of LTC systems to date have focused primarily on typology and financing perspectives, with insufficient attention given to the systemic characteristics that facilitate integrated LTC delivery, as well as the potential challenges of such an approach. Literature specifically focusing on comparing the LTC systems of WPR countries was also lacking, while it could be an important source of information for countries in the region who benchmark each other.

Literature focusing on enabling system-level factors to achieve integrated LTC has mainly come from European countries. After introducing the ICARE4EU project co-funded by the European Union’s Health Programme 2008–2013 [33], management of multimorbidity, which includes frailty and functional disabilities of older people, gained wide interest among the European countries. Various approaches in European countries have been explored, and studies on financing mechanisms to promote integrated care have suggested that single payment mechanisms may not be sufficient to align incentives of fragmented providers, although blended systems of different payment mechanisms may create an adequate incentive [[34], [35], [36]]. After developing the European framework to assess the integrated LTC system [23], country case studies focusing on the assessment of each country had been reported as WHO publications, but efforts to analyze and compare countries to explore the system-level enablers or barriers of integrated care had been rarely conducted. Several recent studies stressed the importance of accountability mechanisms, information systems, and fragmented payment systems, as well as factors such as local leadership in a decentralized setting [[37], [38], [39]]. A review focusing on high-income countries suggested that in order to make micro-level integrated care strategies such as case management or multidisciplinary team work, a whole system approach incorporating linkage between organizations and creating collective accountability should be accompanied [16]. However, none of these studies have specifically focused on WPR countries.

This study aims to address this gap by examining the LTC systems of five selected WPR countries. Using an adapted WHO framework, we analyze key systemic features—governance, financing, workforce, service delivery, information/monitoring & evaluation, and innovation & research—to identify the enablers and challenges for delivering integrated care.

## 2 Methods

### 2.1 Country and area selection

This study incorporates a cross-country comparative analysis using a defined analytical framework (see Supplementary S1). The countries for this comparative analysis were purposefully selected to ensure a robust examination of LTC systems within the WPR, a region where comparative LTC system research focusing on integrated care enablers has been limited despite rapid population aging. Our selection process began by identifying WPR countries with established public LTC systems. Referencing the 2023 progress report for the UN Decade of Healthy Ageing, 11 countries were reported as having established LTC systems out of a total of 32 WPR countries. To focus on countries with more mature systems suitable for in-depth comparative analysis, we further refined this list to include only those five countries—Australia, Japan, New Zealand, the Republic of Korea, and Singapore—that had also reported the presence of their LTC systems in the 2020 baseline report for the UN Decade of Healthy Ageing [40].

This selection of five countries was further supported by their comparability in terms of socioeconomic context and demographic pressures; all are high-income economies as per World Bank classification and are experiencing advanced population aging (defined as ≥14 % of the population aged 65+). Beyond these similarities, the five selected countries represent valuable constructive diversity. They exhibit varied LTC system archetypes, including different financing models (e.g., tax-based versus social insurance) and governance structures (e.g., centralized versus decentralized elements). This diversity allows for a more comprehensive comparative analysis to identify system-level enablers and barriers for integrated care delivery in varied contexts that face common demographic challenges, making them important reference points for other nations in the region. A detailed account of this stepwise country selection process, including the multi-stage filtering logic and rationale, is provided in Supplementary S2.

### 2.2 Framework

This study employs the World Health Organization's (WHO) 2021 "Framework for countries to achieve an integrated continuum of long-term care" as its foundational analytical lens [10]. This framework [10] was preferred over an earlier WHO Regional Office for Europe version due to its comprehensive system-level perspective and explicit alignment with LTC policy objectives [23,33]. It was also preferred over other integrated care frameworks that are not specifically focused on LTC, such as those centered on primary care [21] or populations with multimorbidity in a broader sense [22]. While the WHO (2021) framework provides an excellent overarching structure for LTC systems, we adapted it to specifically evaluate the institutional enablers of integrated LTC for robust cross-country comparison. This adaptation involved a two-stage process.

First, to sharpen the focus on integration, we systematically extracted checklist items from the original WHO (2021) framework (Annex 1) [10] that directly pertain to the key concepts of 'integrated care' at the macro (national policy and system design) and *meso* (organizational and inter-organizational) levels. Second, recognizing that the WHO (2021) framework [10], even when filtered for integration-related items, offers limited detail on some specific meso‑level mechanisms and operational enablers critical for effective integration, we supplemented selected WHO items with components of the SELFIE framework for integrated care [22]. Despite its original focus on multimorbidity, the SELFIE framework provides valuable constructs for understanding the 'how' of integration, such as inter-organizational collaboration, supportive leadership, and financing mechanisms that promote coordination [22]. We screened SELFIE items for their relevance to LTC and their ability to assess these critical enablers for integrated LTC.

This systematic process resulted in a modified analytical framework, presented as a consolidated checklist, designed to assess key enablers of integrated LTC. This checklist spans the six core system components of the WHO framework: governance, sustainable financing, information, monitoring & evaluation systems, workforce, service delivery, and innovation & research. The complete derivation procedure for this modified framework, including the selection and supplementation criteria, and the final consolidated checklist used for the analysis in this manuscript, are detailed in Supplementary S1.

### 2.3 Search strategy

For this review, a comprehensive literature search was conducted using Google, Google Scholar, MEDLINE (PubMed), and official government websites to identify information pertinent to the components of our modified analytical framework's checklist. Additionally, other sources of grey literature, including the WHO database, official government publications, and relevant web pages, were examined. We use documents written in English only for Australia, New Zealand, and Singapore, for Japan and the Republic of Korea, where English is not the official language, literature in English, Japanese, and Korean was also reviewed. A summary table of each country’s LTC system, based on the modified framework, was reviewed for accuracy and completeness by country expert co-authors. Although not the primary focus of this study, which centers on integrated LTC, information helpful for understanding the LTC systems of the included countries is provided in Supplementary S3 for reference.

## 3 Results

### 3.1 Governance

The governance structures underpinning integrated LTC varied significantly across the five countries (Table 1). All five countries demonstrated political commitment to integration through various legislative and strategic documents (GOV2). Australia’s 2022–2025 Aged Care Reform Roadmap emphasizes better integration of health and aged care services [41], now embedded within the new *Aged Care Act 2024* [42]. Japan’s Long-Term Care Insurance Act (1997), alongside national strategies such as the 2015 Integrated Community Care System and the 2019 Dementia Policy Framework, provides foundational support for integration [43,44]. Korea recently passed an 'Integrated Community Care Act' (effective March 2026) and includes integration in its 3rd Basic Plan for LTC and various pilot programs [[45], [46], [47]]. In New Zealand, the Healthy Ageing Strategy (2016) serves as the guiding framework for older persons’ care, encouraging integrated models rather than a standalone LTC plan [48]. Singapore’s Long-Term Care Act (2019) and Action Plan for Successful Ageing (2023) outline legal and strategic priorities [49], with national reforms like Healthier SG embedding LTC integration within broader health strategies [50].

**Table 1:** Governance characteristics for the integrated long-term care system across five countries.

	Australia	Japan	Korea	New Zealand	Singapore
**GOV1:** Designated Lead Coordinating Body/Agency for LTC with a Clear Mandate for Integration	* DoHAD oversees both health and LTC policy [52] * No agency bridges health and social care [52]	* MHLW oversees LTC [44] *Municipalities act as insurers with national mandates [44]	* MOHW and National Health Insurance Service (NHIS) co-lead LTC [51] * Historic fragmentation from separate NHI & LTCI systems persists [51]	* Health NZ delivers services [48] * MOH oversees regulation; integration efforts are ongoing following the DHB reform [48].	* MOH oversees LTC [53] * Agency for Integrated Care (AIC) manages community care coordination across sectors [54]
**GOV2:** Explicit Political Commitment, Supported by Legislation or National Strategic Plans, for Integration	* Aged Care Act 2024 is rights-based legislation that outlines objectives of the aged care system, including integrated care[42] * 2022–2025 Aged Care Reform Roadmap highlights integration, but implementation is uneven [41].	* LTCI Act (1997) and reforms promote integrated community care [43,44].	* Integrated Community Care Act (2024) and LTC strategic plans mandate integration [[45], [46], [47]]	* The Healthy Ageing Strategy (2016) guides integrated care in the absence of an LTC-specific plan [48]	* LTC Act (2019) and Action Plan (2023) outline integration priorities [49]
**GOV3**: Formal Mechanisms for Shared Accountability and Collaborative Decision-Making	* Centralized governance [52] * Quality standards exist, but limited shared accountability (i.e. ACQS) [95,96]	* Decentralized financing structure for both NHI & LTCI [43,44] fosters accountability at the municipalities level.	* Formal inter-sectoral mechanisms limited at the municipal level [47,57]	* Health NZ created for unified delivery [48,55] * Shared governance still evolving [48,55]	* Cluster-based planning under the Ministry of Health (MOH) promotes shared accountability [56].
**GOV4**: Established Processes for Engaging Service Users, Carers, and Community Representatives in Planning, Monitoring, and Evaluation	* Several representative councils provide advice on policy[97]	* Community groups active locally [59] * Limited strategic user involvement [59]	* No established national mechanisms for user involvement in planning or M&E [57]	* Code of Expectations (2022) mandates community engagement in health and LTC [58]	* Community input occurs via service-level activities; limited strategic engagement [98,99]

The structure of lead agencies responsible for LTC and promoting integration (GOV1) differed. In Japan and Korea, health and welfare functions are consolidated at the central ministry level, with Japan's Ministry of Health, Labour and Welfare (MHLW) [44] and Korea's Ministry of Health and Welfare (MOHW) leading LTC policy [51]. New Zealand integrates LTC into its general health system, managed by the Ministry of Health for policy and regulation, and Health New Zealand (Health NZ) for operational delivery [48]. Australia's Department of Health, Ageing and Disability (DoHAD), under the federal health ministry, is the primary agency for both health and LTC policy, though no specific agency is mandated to bridge health and social care [52]. Singapore, distinctively, established the Agency for Integrated Care (AIC) as a specialized coordinating body that works closely with the Ministry of Health (MOH) [53]. The AIC's mandate explicitly includes community-based service planning and provider support to foster integration [54].

The locus of accountability for delivering integrated care and the mechanisms to support this (GOV3) presented further contrasts. Accountability is centralized at the federal or national level in Australia and New Zealand. In Australia, the Commonwealth government holds sole responsibility for aged care decision-making [52]. New Zealand's system is also highly centralized, particularly under Health New Zealand, and it lacks formal national-level mechanisms to enforce shared accountability or collaborative decision-making within the LTC sector [48,55]. Conversely, Japan assigns accountability to the municipal level, a system reinforced by its decentralized financing structure for both national health insurance and LTCI [43,44]. Singapore implements accountability through its MOH-overseen integrated regional health clusters, which feature structured planning mechanisms to support inter-agency coordination and performance reporting [56]. Korea’s new 'Integrated Community Care Act' legally designates municipalities as the accountable body for implementing integration once effective in March 2026; however, formal mechanisms to ensure this shared accountability are yet to be established [47,57].

Finally, processes for engaging service users, carers, and community representatives in strategic-level LTC planning, monitoring, and evaluation (GOV4) were generally found to be insufficient, with New Zealand being a notable exception. Mandated by the Pae Ora (Healthy Futures) Act 2022, New Zealand requires health entities, including LTC services, to engage with communities as per a Code of Expectations, and Te Whatu Ora has established standardized payment rates for consumer involvement [58].

### 3.2 Financing

The financing systems across the five countries generally presented significant barriers to integrated LTC (Table 2). This was evident in the limited horizontal integration of funding between health, LTC, and other social protection schemes; the often fragmented or underdeveloped vertical integration mechanisms for coordinating budgets for integrated LTC across different levels of government; and a scarcity of payment models designed to incentivize inter-sectoral collaboration. Singapore, however, demonstrated emerging progress in some of these areas.

**Table 2:** Financing characteristics for the integrated long-term care system across five countries.

Section Topic	Australia	Japan	Korea	New Zealand	Singapore
**FIN1**: Eligibility and Funding Structure of Public LTC Systems and Links to Health Financing	* The public LTC system operates on needs-based eligibility criteria but is not structured for integration or mandatory coordination with healthcare system [52]	* Public LTCI is operated based on needs assessment-based eligibility criteria, but it is not designed to be integrated or to mandate coordination with health insurance schemes [43,44,59]	* Public LTCI is operated based on needs assessment-based eligibility criteria, but it is not designed to be integrated or coordinated with health or other social protection schemes [46,51,60]	* Public LTC system operates on needs-based eligibility criteria, and funding for health and LTC is operated within a unified health budget at the national level [48,55]	* LTC is not funded through a separate insurance pool. MOH and RHS clusters finance services like nursing homes, integrating LTC into broader health budgets, similar to post-acute care [100,101]
**FIN2:** Mechanisms for Coordinating Budgets Across Ministries, Insurance Schemes, or Administrative Levels	* LTC funded federally; healthcare predominantly funded by states with limited coordination [27,62]	* LTCI co-financed by the central (25 %), prefectural (12.5 %), municipal (12.5 %) governments; no joint budgeting with the health sector [43,44]	* No cross-ministry or inter-scheme coordination for LTCI and NHI budgeting [46,60,64]	* Since 2022, Central funding through Health NZ has enabled integrated financing across services [48]	* MOH allocates budgets through regional clusters for integrated LTC through three regional health clusters, enabling integrated service planning across care levels [61].
**FIN3**: Financial Incentives or Payment Models that Promote Service Integration Across Health and LTC	* Payments remain siloed [27,62]. * Aged care lacks incentives for integration [27] * In new Support at Home program, care management services to be funded separately from service delivery[63]	* Separate fee schedules used; no bundled or pooled payment for integration [43,66]	* Volume-based reimbursement dominates; pilots lack defined cross-sector payment models [57,64]	* No financial incentives for integration under the current funding structure [48]	* Bundled payment pilots introduced; capitation and pay-for-performance (P4P) models being explored [65].

Regarding the horizontal integration of public LTC financing with health and other social protection systems (FIN1), explicit design for such integration was largely absent. In Japan, the public LTCI, while based on needs assessment, is not structured to be integrated or to mandate coordination with health insurance schemes [43,44,59]. Similarly, Korea's public LTCI operates with needs-based eligibility but is not designed for integration or coordination with national health insurance or other social protection schemes [46,51,60]. In contrast, funding for health and LTC in New Zealand is operated within a unified health budget at the national level[48,55].

The vertical financial coordination for integrated LTC, particularly how centrally managed and often fragmented funding streams (as identified in FIN1) translate to the local or implementing body level to support integrated service delivery (FIN2), also revealed significant limitations in most countries. Given the general lack of horizontal integration of health and LTC funding at the central level in most reviewed nations (excluding New Zealand), the capacity for local entities to coordinate or pool these distinct budgets for integrated LTC becomes crucial. Singapore is an interesting exception. Singapore’s MOH centrally manages LTC funding, allocating resources through three regional health clusters whose governance structure allows for integrated service planning across various care levels [61].

The use of specific payment models or financial incentives to encourage inter-sectoral collaboration and integrated LTC delivery (FIN3) was rare. In Australia, payments for aged care and healthcare are siloed, reflecting their separate funding systems, with the major aged care funding model does not yet provide incentives for implementing integrated care [27,62]. Nonetheless, a new Support at Home program will provide separate pooled funding for care management services of in-home care [63]. In Korea,volume-based reimbursement dominate both healthcare and LTCI home and community-based services, rewarding service volume rather than inter-sectoral coordination, and specific payment models to foster such collaboration are not detailed or implemented [57,64]. New Zealand also has no explicit financial incentive programs, such as pay-for-performance or bundled payments, to promote collaboration between health and social care providers, with funding and provider payments handled within its unified health budget [48]. Singapore again stands as an exception, having initiated bundled payment pilots from 2023 to encourage care coordination across providers and exploring capitation and selective pay-for-performance elements [65]. Another interesting case is Japan. Japan primarily employs a volume-based reimbursement for its LTCI services and does not utilize direct payment models for integrated care such as bundled payments or pooled budgets with healthcare; its payment systems for health and LTC are managed separately [43,66]. Nevertheless, a distinct financial mechanism exists where municipalities, as LTC insurers, receive performance-based subsidies linked to quality assurance efforts guided by Plan-Do-Check-Act (PDCA) cycles [44,67]. While this system is primarily aimed at overall quality improvement, it may indirectly incentivize municipalities to adopt more integrated approaches to care delivery as a means to achieve better LTC outcomes.

### 3.3 Workforce

The development of a workforce equipped with the necessary interdisciplinary skills, collaborative competencies, and clearly defined roles required for effective integrated LTC delivery was generally found to be in its early stages across the five countries (Table 3).

**Table 3:** Workforce characteristics for the integrated long-term care system across five countries.

Section Topic	Australia	Japan	Korea	New Zealand	Singapore
**WRK1:** National or Regional Workforce Strategy for Interdisciplinary Skills and Integrated Care Roles	* No national workforce strategy focused on integrated or interdisciplinary LTC roles [52]	* Care manager role promoted; inter-professional skills development supported [69]	* No comprehensive national strategy; integration addressed in recent legislation but not implemented [47,64]	* No targeted workforce strategy for LTC integration [48]	* No national strategy for integrated LTC roles [68].
**WRK2:** Education and ongoing training on integrated LTC, inter-professional collaboration, and person-centered approaches to care	* Formal education on interdisciplinary LTC remains limited [52,102]	* Joint education programs underdeveloped; mostly sector-specific [69]	* Training for care managers varies across pilot sites; no national standard [57].	* Some advanced care certificates include integrated care skills [103]	* No formal interdisciplinary training curriculum exists [73]
**WRK3:** Defined and Regulated Roles Supporting Coordination Across Health and Social Care Sectors	* Assessment teams support planning [70] * No national coordinator role [52] * Care partner roles have recommended rather than mandatory qualifications	* Care managers are legally defined for inter-sectoral coordination [43,44]	* Piloted care roles exist; formal definitions lacking [46]	* Roles remain service-specific; no integrated job definitions [71]	* Coordination tasks handled informally; roles not credentialed [72]

Specific national or regional workforce strategies explicitly designed to cultivate skills for integrated LTC (WRK1) were largely absent. Australia does not have an overarching workforce strategy for interdisciplinary or integrated care [52]. Similarly, New Zealand lacks a dedicated national strategy targeting the LTC workforce for integrated care [48], and Singapore has no national workforce plan specifically for integrated LTC roles [68]. In Korea, while a single, comprehensive national strategy is currently missing, its new Integrated Community Care Act provides a legislative basis for future policies and training for specialized integrated care personnel [47,64]. Japan was an exception, with its LTC workforce strategy promoting skill development in care coordination and inter-professional collaboration, particularly for care managers [69].

The establishment of clear definitions and supportive regulations for new or evolving professional roles that facilitate integration across health and social sectors (WRK3), such as care coordinators or navigators, varied. Japan legally recognizes the care manager role, designed to bridge medical, welfare, and community services and play a key part in individual care planning and inter-sectoral coordination [43,44]. In contrast, Australia and New Zealand utilize assessment teams for initial care planning and referrals, with ongoing case management delivered by aged care providers, but neither has an officially regulated integrated care navigator role bridging health and social services on a system-wide basis [52,70,71]. Korea has piloted some new roles like care coordinators, but these are not nationally defined or regulated within its LTCI scheme, and job roles tend to remain pilot-specific [46]. Similarly, in Singapore, roles such as care coordinators or navigators are not legally defined or credentialed, though social workers may informally perform coordination tasks in pilot projects [72].

Formal education and continuous training programs that include joint modules or specific content on integrated LTC, inter-professional collaboration, and person-centered care for complex needs (WRK2) were also limited. Australia and Japan reported that standardized pre- and in-service education programs jointly developed across sectors remain limited [52,69,102]. Singapore also lacks a formal interdisciplinary curriculum on integrated LTC or care coordination [73]. Korea has no standardized national education program for care managers, with such training delivered irregularly and at the discretion of local governments within pilot project areas [57]. In New Zealand, some advanced care certificates included integrated care skills but this certificate is not mandated [103].

### 3.4 Service delivery

The establishment of service delivery mechanisms that actively foster integrated LTC, such as standardized assessment protocols linked to integrated care planning and formalized integrated care pathways, showed varied development across the five countries (Table 4).

**Table 4:** Service delivery characteristics for the integrated long-term care system across five countries.

Section Topic	Australia	Japan	Korea	New Zealand	Singapore
**SRV1:** Assessment Tools Used to Determine Needs, Eligibility, and Care Planning Across Sectors	* National Standardized Assessment Form (NSAF) used [70] * Limited effect on integration [70]	* LTCI uses standardized eligibility assessments [43,44]. * Recent national policy improving coordination [69,104].	* Integrated assessment tool for health and LTC sector being piloted [46,57].	* Single assessment system lacks integration focus [71]	* AIC uses standardized Activities of Daily Living (ADL) tools to support integrated planning [105]. * Integrated Community Care Provider (ICCP) conducts standardized care assessments to identify needs and develop LTC plans [106]
**SRV2:** Care Pathways and Protocols that Enable Service Continuity and Coordination Across Providers	* Initiatives exist, but fragmented service governance hinders integration [52]	* CGSCs and care managers facilitate local coordination; but coordination remains voluntary [43,44,104].	* Protocols vary by pilot sites to enhance regional responsiveness; implementation remains voluntary [46,57].	* Standardized assessments support care planning via coordinators [107]	* Bundled care and transitional services promote care continuity [65,108] * ICCP coordinates a full suite of services (AAC, SCC, HPC+, HT) [106]***

*** Active Ageing Centre (AAC), Senior Care Centre (SCC), Enhanced Home Personal Care (HPC+) and Home Therapy (HT) Providers.

All countries reported using standardized person-centered assessment protocols (SRV1), but their role in fostering integrated care differed. In Australia, every client undergoes a multi-dimensional needs assessment using a National Standardised Assessment Form (NSAF); however, this assessment was reported to hardly lead to enhancing integrated care provision [70]. Similarly, while New Zealand employs a single, standardized assessment process nationwide, it reportedly does not enhance integrated care provision. Korea, which historically used standardized needs assessment tools primarily for LTCI eligibility and grading, has developed and is currently piloting a more comprehensive assessment tool designed to cover both medical and social care needs. Japan has nationally standardized LTC eligibility levels and care management protocols, with recent national policy promoting improved care management quality to better coordinate with medical and community-based services. Singapore’s Agency for Integrated Care (AIC) utilizes standardized ADL-based assessments that explicitly support person-centered care planning, and these assessment results are used to facilitate integrated care coordination across health and social providers [106].

The implementation of standardized processes and procedures for creating integrated LTC pathways, including clear transition policies and case/care management protocols (SRV2), was also inconsistent. Australia has initiatives such as the Transition Care Program (TCP) for post-hospital discharge support, and strengthening case management and formal referral protocols between hospitals and aged care providers is an ongoing effort; however, gaps in seamless care persist. In Japan, Community-based General Support Centers (CGSCs) and care managers support local-level coordination across sectors, but the actual coordination often remains largely voluntary. Korea, alongside its standardized needs assessment tools and use of care managers, intentionally decentralized implementation protocols in pilot areas to reflect local contexts, where actual coordination also tends to be voluntary. New Zealand reported having no single national protocol for integrated LTC pathways. In contrast, Singapore employs coordinated service packages across acute and community care through Integrated Community Care Providers (ICCP) [106].

### 3.5 Information, monitoring & evaluation

The development of robust information, monitoring & evaluation (IM&E) systems to support person-centered, coordinated LTC, including integrated information platforms, relevant minimum data sets (MDS), and secure information exchange protocols, showed ongoing efforts but also significant challenges across the five countries (Table 5).

**Table 5:** Information, Monitoring & Evaluation (IM&E) characteristics for the integrated long-term care system across five countries.

Section Topic	Australia	Japan	Korea	New Zealand	Singapore
**IME1:** National/Regional Strategies to Integrate and Link LTC and Health Information Systems for Person-Centered, Coordinated Care	* The Australian Digital Health Agency is linking aged care providers to My Health Record for access to resident health data, including discharge summaries. By mid-2024, 80 % of facilities are expected to be connected [74,75].	* The 2018 and 2021 LTCI law revisions introduced PDCA-based planning and promoted the development of LTC data infrastructure to support care coordination [44,67].	* New 'Integrated Community Care Act' mandates the establishment and operation of an integrated information system to support coordinated care [47].	* New Zealand is developing Hira, a national health information platform (by 2026) that will integrate data from fragmented systems into a single record. It will include health, well-being, and LTC data to support coordinated care [48]	* AIC and MOH support digitization and interoperability of LTC documentation [76]. * Secure, consent-based exchange of information is being scaled across providers [76].
**IME2:** Defined Minimum Data Set (MDS) or Monitoring Tools that Measure Integration and Coordination in LTC Systems	* Aged Care NMDS progressively being implemented, but it lacks indicators on integrated service delivery [77].	* LTC data systems such as CHASE collect functional and clinical indicators. However, standardized measures of care transitions or coordination experience are not fully developed [67].	* Some performance indicators relevant to integrated care—such as the frequency of coordination meetings, the number of linked services per client, and the quality of case management—are being collected as part of pilot projects [57].	* InterRAI in New Zealand functions as an MDS, capturing clinical and LTC status indicators (e.g., ADL/IADL) to support integrated care, though it lacks care process data such as care transitions or coordination experiences [109]	* No national LTC-specific MDS exists; current indicators are primarily administrative [76,78]. * Integration-related quality or outcome metrics are not yet standardized [76,78],
**IME3:** Data-Sharing Mechanisms for Integrated Case Management Across Institutions and Sectors	* My Health Record is the leading secure platform for sharing patient data. As an opt-out system, individuals consent by participation, and providers can view and upload information [74,75]	* LTC databases (CHASE, VISIT) introduced since 2018, but nationwide interoperability, real-time data exchange, and full integration with health data systems remain limited [67].	* MyHealthWay platform (2021) initiative planning to integrate various health data for PHRs [79], but full integration between health and LTC data between providers remains limited.	* There is presently no single, unified electronic system for sharing client information across all health and social care providers in New Zealand [48]	* The National Electronic Health Record (NEHR) supports secure data exchange; LTC integration is ongoing [76] * RHS clusters test consent-based information-sharing protocols to support care coordination across health and LTC providers [76]

All countries reported national strategies aimed at integrating and linking LTC and health information systems to support coordinated care (IME1). Australia’s Digital Health Agency is actively working to connect residential aged care providers to My Health Record, with a goal for 80 % of facilities to be linked by mid-2024 to enable access to up-to-date health information [74,75]. Japan’s 2018 and 2021 LTCI law revisions promoted the development of LTC data infrastructure to support care coordination [44,67]. Korea’s new 'Integrated Community Care Act' mandates the establishment and operation of an integrated information system [47]. While New Zealand’s current information systems are fragmented, the country is developing 'Hira,' a new national health information platform expected around 2026, which aims to integrate health, well-being, and community care information, including LTC service usage [48]. In Singapore, the AIC and MOH support the digitization and interoperability of LTC documentation, with secure, consent-based exchange of information being scaled across providers [76].

However, the establishment of defined Minimum Data Sets (MDS) for LTC that include specific indicators relevant to integrated service delivery (IME2) was generally less developed. Australia initiated an Aged Care National Minimum Data Set (NMDS) following a Royal Commission’s recommendation, but it lacks indicators on integrated service delivery [77]. Japan’s LTC data systems, such as CHASE, collect functional and clinical indicators, but standardized measures of care transitions or coordination experience are not yet fully developed [67]. Korea collects some performance indicators relevant to integrated care, like frequency of coordination meetings and number of linked services per client, as part of pilot projects [57]. While InterRAI functions as an MDS in New Zealand, capturing clinical and LTC status indicators (e.g., ADL/IADL) to support integrated care, it lacks care process data such as care transitions or coordination experiences [109]. Similarly, Singapore has no national LTC-specific MDS; current indicators are mostly administrative, and integration-related quality or outcome metrics are not yet standardized [76,78].

Regarding established protocols and technological infrastructure for the secure exchange of client information between different health and social care providers (IME3), efforts were underway but faced challenges. Australia’s My Health Record provides the main secure infrastructure for sharing patient information through an opt-out system with implicit consent [74,75]. Japan has introduced LTC databases (CHASE, VISIT) since 2018, but nationwide interoperability, real-time data exchange, and full integration with health data systems remain limited [67]. Korea’s MyHealthWay platform initiative (2021) plans to integrate various health data for Personal Health Records (PHRs), but full integration of data between health and LTC providers remains limited [79]. New Zealand currently has no single, unified electronic system for sharing client information across all health and social care providers [48]. In Singapore, while National Electronic Health Record (NEHR) supports secure data exchange and the integration of LTC data is in progress, RHS clusters are also testing consent-based information-sharing protocols to strengthen coordination between health and LTC services [76].

### 3.6 Innovation & research

The promotion of innovation and research, particularly concerning digital technologies to facilitate integration and dedicated strategies for evaluating integrated LTC models, showed varied levels of policy support and development across the five countries (Table 6).

**Table 6:** Innovation & Research characteristics for the integrated long-term care system across five countries.

Section Topic	Australia	Japan	Korea	New Zealand	Singapore
**INR1:** Adoption of Digital Tools and Innovative Technologies to Support Integrated LTC Delivery	* The push to connect aged care to My Health Record (discussed above) is a centerpiece, enabling electronic communication and information sharing [74,75]	* 2021 LTCI law revisions fostered the development of further data infrastructure for promoting the integration of health care and LTC systems [44,67].	* The national LTC policy, as well as the new act, supports digital innovation (e.g., smart home care, remote consultations); however, digital systems for sectoral coordination are still in the early stages [46,47].	* No dedicated innovation program exists for LTC, which lags behind other health sectors in technology adoption. Limited investment has resulted in a lack of tools specific to LTC, such as telehealth or innovative care systems [48].	* The Community Care Digital Transformation Plan (CCDTP) promotes interoperable systems, including secure messaging and telehealth, to support coordination across hospitals, primary care, and community-based LTC [76]
**INR2:** National/Regional Research, Pilots, or Evaluation Mechanisms to Support Innovation in Integrated LTC	* ARIIA, established in 2021, is a federally funded research and innovation incubator and implementation hub[110] * Limited specific innovation focus on LTC integration[80]	* Japan institutionalized PDCA cycles for municipalities and long-term care (LTC) providers, supported by national data systems (e.g., CHASE, VISIT). Municipalities receive performance-based subsidies and coordinate care, supporting quality assurance and local model development [44,67].	* Some government-funded research supports pilot evaluations, but no dedicated research or evaluation for innovation exists nationally [57].	* New Zealand does not have a specific national strategy or funding program focused exclusively on long-term care research and innovation. Long-term care research is embedded mainly on the broader health research framework [48]	* Although no dedicated national research strategy exists, RHS clusters operate under performance-based governance that incentivizes innovation in integrated long-term care (LTC) delivery and evaluation [98,111].

National or regional policies fostering the development and adoption of digital information technologies—such as telehealth, shared electronic records, and mobile health applications—specifically to facilitate communication, information exchange, and coordination in integrated LTC (INR1) were present in most countries, though with differing focuses and stages of development. Australia's push to connect aged care providers to the My Health Record system is a central initiative aimed at enabling electronic communication and information sharing [74,75]. Japan’s 2021 LTCI law revisions fostered further development of data infrastructure to promote the integration of healthcare and LTC systems [44,67]. Korea’s national LTC policy and its new Integrated Community Care Act support digital innovation, including smart home care and remote consultations, although digital systems specifically for sectoral coordination are reported to be in their early stages [46,47]. In Singapore, the Community Care Digital Transformation Plan (CCDTP) promotes interoperable systems, including secure messaging and telehealth, to support coordination across hospitals, primary care, and community-based LTC [76]. In contrast, New Zealand reported no specific innovation program devoted solely to LTC technology [48], noting that the LTC sector has lagged behind other health sectors in adopting new technologies due to limited targeted investment.

Regarding national or regional strategies or funding mechanisms to promote research and innovation specifically in integrated LTC models, including their evaluation for effectiveness, cost-effectiveness, and scalability (INR2), dedicated efforts were less common. Australia recently bolstered its LTC research infrastructure with the creation of Aged Care Research & Industry Innovation Australia (ARIIA) in 2021, but specific innovation focus on LTC integration is limited [80]. Japan has institutionalized PDCA cycles for municipalities and LTC providers, supported by national data systems; while this system primarily focuses on quality assurance, it offers a potential platform for structured experimentation and model development at the local level, with municipalities receiving performance-based subsidies and coordinating local care integration [44,67]. Korea has some government-funded research supporting pilot evaluations [57], but no dedicated national research or evaluation framework for innovation in integrated LTC exists. New Zealand does not have a specific national strategy or funding program focused exclusively on LTC research and innovation; such research is largely embedded within the wider health research framework [48]. Singapore lacks a dedicated national strategy or funding mechanism explicitly for research and innovation in integrated LTC models [78], but RHS clusters operate under performance-based governance that incentivizes innovation in integrated LTC delivery and evaluation [98,111].

## 4 Discussion

Achieving an integrated LTC system requires fostering coordination among fragmented service providers. This is particularly important in WPR countries, where LTC services are predominantly provided by the private sector. While pursuing an integrated LTC system is often recommended as the goal in WPR countries, this study highlights that the system-level enabling factors are frequently lacking. A key finding in governance was the universal political commitment to care integration across the five WPR countries. However, our analysis reveals significant challenges and variations in translating this political will into effective governance structures capable of fostering integrated LTC, particularly concerning the balance between centralized control and localized accountability.

The structure of accountability for delivering integrated LTC diverged significantly, which is closely linked with the reluctance to decentralize financing responsibilities. While the role of local authorities in service delivery, particularly in reflecting local contexts — such as coordinating health promotion and personal support services at community-level centers — was prevalent in most of the countries studied [[81], [82], [83], [84]], further decentralization of accountability proved difficult. While centralization can promote consistency and address equity concerns, as seen in Australia’s 2012 National Health Reform Agreement which emphasized administrative efficiency and equity [85], it may also limit local flexibility. New Zealand’s current centralized structure under Health New Zealand [48,55], for instance, lacks formal national-level mechanisms to enforce shared accountability specifically between the multiple layers of governance in LTC sector, a context perhaps informed by past experiences where decentralized purchasing based on risk-adjusted fixed budgets led to service access inequities [86]. While Korea is moving towards municipal accountability with its new 'Integrated Community Care Act,' formal mechanisms to support this shift are yet to be established, largely due to the historical fragmentation of its centrally operated health insurance and LTCI.

In contrast, Japan assigns LTC accountability to the municipal level, a system reinforced by its decentralized revenue generation structure for both health insurance and LTCI [43,44]. However, this model may also face challenges, such as regional disparities in insurance premiums arising from decentralized revenue generation paired with centralized service purchasing criteria [87]. In this regard, Singapore’s reform, which introduced decentralized health clusters [56], warrants closer examination in future studies. By selectively implementing decentralized purchasing and allowing beneficiaries the freedom to choose providers, Singapore fostered competition among health clusters, improving the responsiveness of the health-related LTC sector. At the same time, it minimized financial inequities by maintaining centralized revenue generation. Notably, the ability for individuals to choose health-related LTC services across health clusters helped to balance competition with equity considerations [88].

Furthermore, the payment models and financial incentives used in most of the countries studied were rarely designed to encourage inter-sectoral collaboration or integrated LTC delivery. As noted in previous literature, allowing flexible purchasing power alongside integrated funding is considered to enhance coordination [35,36], yet such mechanisms remain uncommon. Theoretically, integrating health and social care funds can create collective accountability between these sectors by enabling more strategic purchasing [16,89]. Since the key outcomes of integrated LTC focus on values like health, cost, and patient experiences, value-based blended payment schemes—such as combining global payments with explicit quality incentives—could better align incentives between providers [89]. Singapore again offers a pathfinder example, having initiated bundled payment pilots and exploring capitation and selective pay-for-performance elements to encourage care coordination [65]. Japan also presents a nuanced case; while it primarily employs an volume-based reimbursement for LTCI services and lacks direct payment models for integrated care like bundled payments [43,66], a distinct financial mechanism exists where municipalities, as LTC insurers, receive performance-based subsidies linked to quality assurance efforts guided by PDCA cycles [44,67]. This municipal-level accountability is further amplified by their fiscal responsibility for LTC insurance, which can involve adjusting local premium rates to cover financial shortfalls. However, a subsequent challenge lies in ensuring these municipal-level drivers effectively translate into collaborative behaviors among the predominantly private service providers. These providers typically operate under a voulme-based reimbursement system, which may necessitate additional local strategies to align their motivations with integrated care goals.

The preceding discussions on governance and financing underscore that effective accountability mechanisms and a shift towards value-based reimbursement are critical for advancing integrated LTC. However, the operationalization of such systems is critically dependent on robust IM&E systems. Specifically, two core components could be essential: first, interoperable information transfer systems that enable seamless communication and data sharing among diverse stakeholders (IME3); and second, a suite of validated performance indicators that can be monitored in real-time to inform value-based payment models and hold systems accountable for achieving desired outcomes of integrated care (IME2). Regarding the first component, the development of interoperable information transfer systems (IME3), our findings indicate that while most of the studied countries are still in the relatively early stages of achieving comprehensive and seamless data exchange specifically for integrated LTC, significant national-level efforts are underway in nearly all of them to advance these capabilities.

However, a perhaps more significant caveat, tied to the second essential component of IM&E systems (IME2), is the general lack of well-defined, person-centered MDS and robust performance tracking indicators for integrated LTC in most of the countries studied. This deficiency in suitable performance indicators is partly due to a broader, ongoing challenge in the global research community to identify and agree upon what specific metrics best capture the successful functioning and impact of integrated LTC initiatives. While several frameworks exist that suggest a broad set of theoretical determinants for integrated care [10,[20], [21], [22], [23]], these frameworks—including the one used in this study—primarily function as tools for evaluating system-level enablers, such as governance, financing, workforce, and service delivery mechanisms. While these structural and process-oriented components are crucial for understanding how integrated LTC is organized and delivered, they do not provide a direct means of assessing whether person-centered integrated service delivery is effectively achieved or how well integrated care translates into improved outcomes for individuals. Developing a more robust approach to evaluating integrated LTC systems requires moving beyond structural assessments and incorporating quantifiable indicators that measure the extent to which care is person-centered at the individual level, such as patient experiences, patient outcomes, as well as system level effects [90].

Notably, a systematic review found that evidence on instruments for measuring integrated LTC remains “vague and limited,” underscoring the difficulty researchers and policymakers face in this area [91]. At present, no universally accepted index or set of indicators exists to capture person-centered integrated LTC across different contexts [92]. Future work that develop and validate specific metrics – for example, patient-reported experience measures focused on care coordination, continuity-of-care indexes, or composite indicators assessing integration of services – to enable more objective comparisons should be further discussed, building on existing examples [93]. Further association of these patient-reported experience measures with other intermediate outcome measures such as the proportion of community-dwelling older adults who experience unmet LTC needs, informal caregiver burden, or LTC institutionalization could be studied. Such indicators would not only enhance empirical assessment but also facilitate cross-country comparability, a key limitation of current evaluation frameworks. Given these challenges, future efforts by international organizations such as the WHO should focus on harmonizing standardized, person-level performance metrics that can capture the impact of integrated LTC policies on individuals’ experiences and outcomes that could be applied to different countries. Existing data sources, such as longitudinal health and retirement studies, could be leveraged and expanded to construct such indicators. By bridging this measurement gap, it will be possible to better assess the effectiveness of LTC systems in delivering truly integrated, person-centered care and provide stronger evidence for policy improvements.

Beyond governance, financing, and IM&E systems, the development of a skilled workforce and the strategic use of innovation are also crucial for realizing effective integrated LTC. However, our findings indicate a significant gap in workforce development specifically tailored for integrated LTC. This general lack of a workforce specifically prepared for integrated care raises critical questions, especially when considered against the backdrop of broader healthcare workforce shortages prevalent in many developed nations. The feasibility of training and sustaining a completely new and distinct cadre of "integrated care specialists" on a large scale is debatable under such circumstances. While enhancing the interdisciplinary skills of the existing workforce is essential, merely adding new specialized roles without addressing systemic workforce capacity issues may not be a sustainable solution. It is in this context that leveraging technological advancements and fostering innovation (as explored in our INR1 findings) becomes paramount. Digital information technologies, including shared electronic health records and telehealth, which most countries are actively pursuing, can play a vital role. However, the mere availability of technology is insufficient; its effective integration into LTC workflows and its capacity to genuinely support a strained workforce require dedicated strategies and ongoing research. However, our findings on national efforts to promote research and innovation in integrated LTC models (INR2) varied, showing further need for consideration in such aspect.

This study highlights that factors favorable to person-centered integrated LTC delivery, such as a whole-system approach that creates accountability mechanisms [16], are often lacking in WPR countries. Reforming such accountability systems presents significant challenges, as it necessitates not only a shared vision [18] among diverse stakeholders—akin to the "triple aim" —but more critically, the alignment of individual-level incentives and motivations for genuine collaboration. The process of fostering this collaboration often resembles a political arena rather than a purely neutral coordination effort [16]. Consequently, the development of ideal LTC systems to support person-centered integrated care must be understood within the broader political economy of healthcare, profoundly shaped by historical path dependencies of ideas, institutions, and interests [94]. This context helps explain why LTC systems in the WPR may diverge from theoretically favorable approaches that have proven successful in other regions.

Despite the limitation of focusing on the major LTC schemes within selected WPR countries, this study makes several significant contributions to literature. It is one of the first to systematically compare LTC systems across multiple WPR countries using the WHO's comprehensive LTC framework as an analytical lens adapted to assess enablers for integrated LTC. Given that the countries included often serve as benchmarks for their neighbors, this research provides a valuable resource for nations in the region contemplating LTC system reforms, thereby enriching a body of literature that has predominantly focused on North American and European contexts. Furthermore, by examining these LTC systems through the specific lens of person-centered integrated LTC, this study offers actionable insights for the analyzed countries to enhance their current systems and provides lessons for other nations aspiring to similar reforms.

## 5 Conclusion

This study of five WPR countries highlights a widespread commitment to integrated LTC. However, translating this aspiration into reality is significantly hampered by underdeveloped system-level enablers, particularly those crucial for establishing robust accountability mechanisms for integrated care.

Effective integration hinges on governance structures that clearly define and assign accountability for coordinating services across fragmented health and social sectors. While political will exists, realizing this through effective central or local/regional accountability frameworks requires not only well-mandated lead agencies but also financing systems that reinforce these responsibilities. Our findings indicate that current financing arrangements—often characterized by a lack of horizontal integration between health and LTC funds, insufficient vertical coordination of budgets to support local integrated care delivery, and payment models that do not incentivize collaboration—frequently undermine efforts to establish such accountability. Furthermore, the operationalization of accountable, value-based integrated care is critically dependent on robust Information, Monitoring & Evaluation systems. Yet, significant challenges persist in achieving seamless information exchange and, more critically, in developing standardized performance indicators that can track the outcomes of integrated care and inform accountability processes.

Addressing these interconnected systemic weaknesses is vital. Strengthening integrated LTC by fostering clear governance and accountability, aligning financial flows and incentives, and building robust data infrastructure is essential. These systemic reforms, by enabling more coordinated, responsive, and measurable care, will, in turn, more effectively support the overarching goal of enabling older adults to age in place within their communities. Future efforts must focus on developing context-specific, whole-system approaches that strategically build these enabling capacities.

## Funding

This research was funded by World Health Organization Regional office for the Western Pacific.

## Research in context

(1) *What is already know about the topic?*
  Achieving ageing in place often relies on providing effective integrated long-term care (LTC), ideally person-centered, to manage complex needs. However, discussions on the specific system components required to achieve effective integrated LTC remain limited. Existing LTC system analyses focus mainly on typology and financing, with insufficient attention to the broader range of systemic characteristics that enable or hinder effective integrated LTC.
(2) *What does this study add to the literature?*
  This study applies an adapted and comprehensive LTC framework to assess the systems of five high-income Western Pacific countries. It examines how specific system features within six dimensions create enablers and barriers to delivering effective integrated LTC.
(3) *What are the policy implications?*
  Policy efforts to advance effective integrated LTC in the Western Pacific Region should prioritize establishing clear accountability mechanisms within governance structures. This requires better coordination of financing through aligned budgets and payment models that incentivize collaboration, alongside developing robust information, monitoring, and evaluation systems with relevant performance indicators. Strengthening workforce capacity for integrated practice and leveraging technological innovation could also be key.

## CRediT authorship contribution statement

**Dongkyu Lee:** Writing – review & editing, Writing – original draft, Methodology, Investigation, Formal analysis. **Soojung Kim:** Writing – review & editing, Writing – original draft, Investigation. **Soonman Kwon:** Writing – review & editing, Supervision, Funding acquisition, Conceptualization. **Sunghun Yun:** Writing – review & editing, Writing – original draft, Project administration, Methodology, Formal analysis, Conceptualization. **Mikiko Kanda:** Writing – review & editing, Project administration, Conceptualization. **Siwon Lee:** Writing – review & editing, Project administration, Conceptualization. **Nicole Sutton:** Writing – review & editing, Investigation. **Paul Ong:** Writing – review & editing, Investigation. **Andy Inder:** Writing – review & editing, Investigation.

## Declaration of competing interest

None declared.

## Appendix Supplementary materials

Image, application 1
