# Supplementary material for: Are long-term care systems aligned with person-centered integrated care? Evidence from the Western Pacific
Source: Health Policy. 2025 Dec;162:105442. doi: 10.1016/j.healthpol.2025.105442 (PMC12624384; doi:10.1016/j.healthpol.2025.105442)
Supplement: Supplementary file 1 [file mmc1.docx]

# Supplementary S1. Deriving a Modified WHO Framework for Cross-country Comparison of Integrated Long-Term Care Systems

Our work is grounded in WHO (2021) framework, *Framework for Countries to Achieve an Integrated Continuum of Long-Term Care (1)*. The WHO framework provides a foundational basis with strong potential for broad applicability. While this framework offers valuable guidance for establishing and strengthening long-term care (LTC) systems, it remains limited as an evaluative tool, particularly for cross-country comparisons assessing the extent to which LTC systems are oriented toward integrated service provision.

The WHO (2021) framework is structured in two parts: the first describes six enablers, referred to as “elements,” and the second provides a checklist of items corresponding to each element, intended to help identify the status of these enablers in integrated LTC provision. For evaluative purposes, reliance on this checklist is essential; however, three key limitations exist. First, many checklist items effectively identify characteristics of general LTC systems but do not specifically address the “integrated” aspect of service provision. Second, the framework provides limited explicit detail regarding meso-level organizational structural features such as inter-agency processes or operational incentives. Third, the checklist comprises eighty-five items, which may be overly extensive for practical application in country-comparison study.

To address these challenges, we implemented a two-step modification process. First, we selectively extracted checklist items from the WHO framework that emphasize care integration. Second, we supplemented these with items from the SELFIE framework (*Sustainable intEgrated chronic care modeLs for multi-morbidity: delivery, Financing, and performance, 2018*) (2), which is conceptually rich and particularly suited for assessing integration mechanisms. Then we refined the overall checklist items to enhance their relevance to integrated LTC provision, drawing upon the SELFIE framework (2018) for guidance. The detailed modification steps are described below.

## Step 1. Extracting ‘Integrated Care’ Components from the WHO Framework

### 1.1. Brief Overview of the WHO Framework (2021)

The framework outlines six core elements essential for strong LTC systems:

1. **Governance:** Leadership, policy, regulation, legal frameworks, stakeholder engagement, and accountability in LTC oversight.
2. **Sustainable Financing:** Revenue generation, fund pooling, and service purchasing to ensure financial protection, equitable access, and system sustainability.
3. **Information, Monitoring, and Evaluation:** Data collection and use for planning, performance measurement, quality improvement, research, and accountability.
4. **Workforce:** Planning, training, recruitment, retention, and management of formal and informal LTC providers.
5. **Service Delivery:** Care models, service types, coordination, quality standards, and organization to meet person-centred needs.
6. **Innovation and Research:** Development and application of new knowledge and technologies to enhance LTC effectiveness and quality.

Central to the framework is WHO’s concept of an integrated continuum of long-term care, emphasizing coordination across health and social sectors. It ensures seamless transitions across care settings (home, community, residential, acute) and collaboration among care roles (prevention, rehabilitation, palliative, acute). Integration spans all care intensities and is delivered timely, combining health and social services holistically. This concept anchors the WHO’s definition of integrated LTC and guides its implementation.

### 1.2. Selection Criteria and Identified Limitations

To adapt the WHO framework for the specific purpose of evaluating and cross-country comparison of integrated LTC systems, the initial step involved systematically extracting components from its Annex 1 checklist, Checklist of key action points for strengthening long-term care systems, from WHO (2021) that directly pertain to 'integrated care' from WHO (2021). The following criteria were applied:

- **Criterion 1: Explicit Terminology** – The item explicitly mentions "integration," "coordination," "collaboration," "intersectoral," or "multisectoral" actions or arrangements.
- **Criterion 2: Health-Social Linkages** – The item focuses on establishing or strengthening linkages, partnerships, or joint working between the health and social care sectors or services.
- **Criterion 3: Continuum of Care** – The item refers to the development or implementation of seamless care pathways, smooth transitions between care settings, or a holistic continuum of care.
- **Criterion 4: Systemic Person-Centeredness** – The item addresses person-centred approaches specifically within the context of service coordination, system design for integration, or inter-provider collaboration.
- **Criterion 5: Macro/Meso Level Relevance** – The item pertains to macro-level (national/system-wide) or meso-level (organizational/inter-organizational) institutional arrangements, policies, or strategies, rather than purely micro-level clinical practices or individual care interactions.

### 1.3. Revised Checklists: More ‘Integrated Care’ Focused WHO Framework

Table 1 presents selected WHO checklist items with an emphasis on integrated care provision. The column "WHO Elements" categorizes each checklist item according to the corresponding domain within the WHO framework. The "Item #" column assigns a unique identifier to each checklist item (e.g., WG1). "Checklist Questions” lists the specific items extracted from the WHO (2021) framework checklist that pertain to integrated care. Finally, the "Rationale for Selections" column provides the justification for the inclusion of each checklist item, based on predetermined selection criteria.

**Table ST1: Selection Criteria and Extracted 'Integrated Care' Components from the WHO (2021) LTC Framework (with identified shortcomings for national evaluation)**

| **WHO Elements** | **Item #** | **Checklist Questions** | **Rationale for Selections** |
| --- | --- | --- | --- |
| 1. Governance | WG1 | "Include long-term care within the portfolio of national and regional or municipal governments with the designation of a dedicated focal coordinating body aiming at reaching leadership and engaging community representatives and other relevant stakeholders." | Criterion 1 (coordinating), Criterion 2 (stakeholders often cross sectors), Criterion 5 (macro/meso) |
|  | WG2 | "Define strategies and actions to facilitate leadership, foster collaboration between health and social systems, and enable alliances to promote more integrated models of long-term care." | Criterion 1 (collaboration, integrated), Criterion 2 (health and social), Criterion 5 (macro) |
|  | WG3 | "Allocate and coordinate budgets across multiple levels of governance to implement an integrated long-term care system." | Criterion 1 (coordinate, integrated), Criterion 5 (macro/meso) |
|  | WG4 | "Promote intersectoral collaboration (health and other government sectors, as well as representatives from private, voluntary and non-profit groups) to build on common goals and to allocate resources." | Criterion 1 (intersectorial), Criterion 5 (macro/meso) |
| 2. Sustainable Financing | WF1 | "Establish a public long-term care financing system with a defined set of eligibility criteria that is used to determine access to and entitlement for a publicly funded range of services that should be available for people, as well as the responsibilities for long-term care, recognizing that this does not need to be a stand-alone financing system, but a predictable financing system for long-term care that is well integrated with other health and social systems. " | Criterion 1 (integrated care), Criterion 2 (implies health/social funding links), Criterion 5 (macro) |
|  | WF2 | "Establish financing mechanisms to ensure equitable use and universal coverage of long-term care and to support integrated care for older people (for example, outcomes-based financing, pay for performance and bundled payments)." | Criterion 1 (integrated care), Criterion 2 (implies health/social funding links), Criterion 5 (macro) |
| 3. Information, Monitoring and Evaluation Systems | WI1 | "Integrate and link long-term care information systems with health information systems to ensure person-centred approaches." | Criterion 1 (integrate), Criterion 4 (person-centred), Criterion 5 (macro/meso) |
|  | WI2 | "Create integrated minimum data sets (clinical outcomes, integrated service targets, composite quality measures) used routinely to support the sharing of and improve the quality of information between all stakeholders." | Criterion 1 (integrate), Criterion 5 (macro/meso) |
|  | WI3 | "Map the number of practitioners working at the interfaces or transitions of care (for example, medical liaisons, social protection worker care managers, nurse discharge managers). " | Criterion 3 (continuum of care), Criterion 5 (macro/meso) |
| 5. Service Delivery | WS1 | “Set up standardized person-centred assessment protocols, including degrees or levels of dependency categories, health criteria assessment to qualify for services, preferences, and older adult and carer needs.” | Criterion 4 (person-centred in coordination), Criterion 5 (meso) |
|  | WS2 | "Define clear processes and procedures to create integrated and person-centred care pathways (transition policies, case prioritization, case and care management)." | Criterion 1 (integrated), Criterion 3 (pathways, transitions), Criterion 4 (person-centred in coordination), Criterion 5 (meso) |
|  | WS3 | "Set up strategies for coordination and communication systems between services providers, health care workers, care receivers and carers, including sharing information between clinicians about patient care." | Criterion 1 (coordination, communication), Criterion 2 (between providers), Criterion 5 (meso) |
| 6. Innovation and  research | WI1 | “Foster the development of digital information technologies to facilitate communication and information exchange among sectors and stakeholders” | Criterion 1 (communication), Criterion 2 (among sectors and stakeholders) |

Applying these criteria to the WHO checklist allows for the identification of foundational elements for an integrated LTC evaluation tool; however, this process also highlights persistent limitations when the extracted content is considered for the nuanced task of evaluating integration at a national level. First, no items correspond to element 4 (Workforce). Unfortunately, the original checklist items under Element 4 do not directly address the provision of integrated care according to the defined criteria. For example, these items include “formulate evaluation mechanisms for current workforce capacity,” “… ensure staff retention…,” and “… ensure gender equity in care provision.” Second, limited focus on meso-level mechanisms. While the WHO framework is comprehensive at a macro (policy) level, it offers less explicit detail on the specific meso-level organizational structures, inter-agency processes, shared protocols, and operational incentives that enable or hinder effective integration between different providers and sectors. These meso-level elements are critical for translating macro-level policies into tangible, integrated service delivery. Third, generality of directives. Many WHO checklist items, while crucial, are framed as broad policy directives (e.g., "Define strategies and actions to facilitate leadership, foster collaboration between health and social systems..."). Such items are excellent for guiding system development but may lack the specificity required to evaluate the *extent, specific mechanisms, or operational effectiveness* of existing integration efforts. The framework outlines *what* national LTC systems should aim for regarding integration, but a more detailed assessment of *how well* these aims are being achieved and through *which specific institutional mechanisms* are necessary. These limitations underscore the need for supplementation to create a more robust evaluative tool for the purpose of evaluating national integrated LTC.

## Step 2. Enhancing the Framework with SELFIE: Criteria for Strengthening Integrated LTC Assessment

To address the identified limitations of the WHO framework for the specific purpose of evaluating national integrated LTC systems, the SELFIE framework offers valuable conceptual and structural enhancements.

### 2.1. Brief Overview of the SELFIE Framework

The SELFIE framework (2018) was developed through extensive literature reviews and expert consultations across multiple European countries, involving patients, informal caregivers, professionals, payers, and policymakers (the "5Ps"). Its multi-level perspective (micro, meso, macro) and detailed consideration of mechanisms that enable integration, such as governance structures, financing models, and service delivery configurations, can significantly enhance the WHO LTC framework for evaluative purposes. Its potential key contributions for this modification include:

- **Holistic, Person-Centred Care:** SELFIE emphasizes understanding individuals with multi-morbidity in their broader environment, prioritizing their unique needs, well-being, and self-management abilities. This positions person-centeredness as a core organizing principle for integrated care.
- **Multi-Level Structure (Micro, Meso, Macro):** SELFIE analyzes integrated care across three levels for six domains adapted from WHO’s health system building blocks. Micro-level focuses on individual care processes and provider interactions. Meso-level covers organizational collaboration, leadership, resource allocation, and payment models translating macro policies into practice. Macro-level involves national policies, legislation, system design, and resource strategies enabling integration. The emphasis on the meso-level is especially important for evaluating institutional LTC, where inter-organizational integration happens.
- **Mechanisms of Integration:** SELFIE specifies concrete mechanisms such as supportive leadership, organizational transparency, formal alliances, and innovative financing models like pay-for-coordination. These elements help operationalize WHO’s broader integration goals by clarifying how integrated care helps move from "what to do" towards "how to structure and assess it."

### 2.2. Proposed Modification Criteria using SELFIE

The SELFIE framework was developed for multi-morbidity care and lacks complete alignment with long-term care systems, especially at the macro level. It is less suited as a standalone tool for evaluating national LTC integration across diverse governance and financing contexts. In contrast, the WHO LTC framework offers a comprehensive system-level foundation, capturing key elements essential for cross-country comparison. Thus, the WHO framework serves as the main structure, with SELFIE integrated to strengthen assessment of meso-level mechanisms and institutional enablers of integration.

The primary goal of framework modification is to enable meaningful cross-country comparisons of how integrated LTC is institutionalized, focusing on the macro and meso levels. Macro-level items assess national policies, legal frameworks, financing, and system-wide strategies, providing context on national commitment and strategic direction. Meso-level items examine organizational arrangements, regional governance, collaboration protocols, resource allocation, training, and shared information systems, showing how policies are implemented, and integration occurs in practice. This dual focus captures both strategic intent and operational mechanisms across different national systems.

Based on the strengths of the SELFIE framework and the identified gaps in the WHO framework for evaluative purposes, the following criteria were established to guide the supplementation process:

- **Criterion 1: Enhancing Evaluation of Mechanisms and Enablers:** The component must enhance the ability to evaluate the *mechanisms, processes, and enabling factors* of integration. This includes specific governance arrangements that foster collaboration, workforce models that support inter-professional teamwork, financial incentives aligned with integration goals, and information sharing protocols that facilitate coordinated care.
- **Criterion 2: Strengthening Systemic Person-Centeredness:** The component should strengthen the evaluation of person-centeredness as a systemic feature embedded within policies, organizational practices, and service design, rather than solely an attribute of individual provider-patient interactions.
- **Criterion 3: Ensuring Cross-Country Comparability:** Items derived or inspired by SELFIE should be suitable for cross-country institutional comparison. This means focusing on structural and process aspects that can be assessed across different national contexts, avoiding overly granular micro-level details that vary extensively and are difficult to compare systematically.
- **Criterion 4: Incorporating Sustainability Aspects:** Where relevant, the component should offer insights into the *sustainability* of integrated care models, reflecting SELFIE's explicit aim to contribute to "Sustainable intEgrated chronic care modeLs". This includes considering factors like stable funding, workforce development, and adaptive governance.

By applying these criteria, the modified framework aims to leverage the strengths of both the WHO and SELFIE frameworks, creating a more nuanced and powerful tool for evaluating national integrated LTC systems.

### 2.3. Enhanced Checklist: Integrating SELFIE Insights into the WHO Framework

The modified framework retains the WHO’s six core elements as its overarching structure, ensuring comprehensiveness and drawing on a globally recognized organizing principle for health and social care systems. For each element, a consolidated checklist is proposed, primarily composed of selected and refined items from the WHO (2021) checklist, supplemented with additional items informed by the SELFIE framework.

The WHO checklist items included are those relevant to evaluating integration at the macro or meso level. Some have been slightly rephrased to align more directly with integrated long-term care evaluation, incorporating conceptual elements from the SELFIE framework, which emphasizes integrated care provision. Additional checklist items were derived from the SELFIE framework. Although SELFIE does not provide a formal checklist, key attributes corresponding to WHO elements, especially at the meso level, were identified and adapted to address conceptual and operational gaps in the WHO framework.

Table 2 presents the finalized checklist. “WHO Elements” denotes the domain from the WHO framework to which each item corresponds. “Item #” assigns a unique identifier to each checklist item (e.g., GOV1). “Checklist Items” provides the specific statements relevant to integrated care. The “Primary Sources and Analytical Justifications” column identifies the origin of each item (e.g., SF for SELFIE-derived items; WG1 or WF1 for WHO items from Table 1) and explains the rationale for its inclusion, based on pre-defined selection criteria.

This enhanced framework significantly improves the capacity of WHO framework to support cross-country evaluations of integrated long-term care. By integrating SELFIE’s meso-level specificity and paying more attention to mechanisms such as payment models, inter-organizational agreements, and shared accountability, the revised checklist becomes a diagnostic tool. It not only identifies whether integration-related policies or structures are in place, but also reveals how they are operationalized, the extent of coordination across sectors, and the institutional dynamics shaping care delivery. This dual-layered approach, merging the structural breadth of WHO with the operational depth of SELFIE, offers a more comprehensive and actionable instrument for assessing national LTC systems.

**Table ST2: Consolidated Checklist for Evaluating National Integrated LTC (Macro/Meso Level)**

| **WHO Elements** | **Item #** | **Checklist Questions** | **Primary Sources**  **and Analytical Justifications** |
| --- | --- | --- | --- |
| 1. Governance | GOV1 | Is there a designated national and/or regional lead coordinating body/agency for LTC with a clear mandate that includes fostering integration between health, social, and other relevant sectors? | WG1, Assesses high-level leadership and coordination structures for integrated LTC |
|  | GOV2 | Is there explicit political commitment, supported by legislation or national strategic plans, that enables and actively promotes the integration of LTC services across different sectors and provider types? | WG2, Evaluates institutionalized collaboration and shared responsibility at the governance level. |
|  | GOV3 | Are there formal mechanisms (e.g., inter-ministerial committees, joint task forces) for shared accountability and transparent, collaborative decision-making between health and social care sectors in the governance of integrated LTC? | WG4, Assesses the policy and legal enabling environment that promote intersectoral collaboration |
|  | GOV4 | Are there established processes for engaging service users, carers, and community representatives in the planning, monitoring, and evaluation of integrated LTC policies and services at a strategic level? | WG4, Assesses systemic inclusion of stakeholder perspectives in governance. |
| 2. Sustainable Financing | FIN1 | Is there a public LTC financing system with defined eligibility criteria that is designed to be integrated or coordinated with health and other social protection financing systems? | WF1, Evaluates the integration of funding streams to support seamless care (revenue raising). |
|  | FIN2 | Is there a mechanism for allocating and coordinating budgets across multiple levels of governance (national, regional, local) or between different ministries (e.g., health, social affairs) to implement integrated LTC systems? | WG3, Assesses inter-sectoral and inter-governmental budgetary coordination (pooling). |
|  | FIN3 | Are there specific payment models or financial incentives (e.g., bundled payments, pay-for-coordination, population-based budgets with risk adjustment, pooled budgets) implemented at a national or regional level to encourage inter-sectoral collaboration and integrated LTC delivery? | WF2, Assesses the use of financial levers to promote integration (purchasing). |
| 3. Information, Monitoring & Evaluation (IM&E) Systems | IME1 | Are there national/regional strategies or plans to integrate and link LTC information systems with health information systems to support person-centred, coordinated care? | WI1, Evaluates strategic intent for integrated information infrastructure. |
|  | IME2 | Is there a defined minimum data set for LTC that includes indicators relevant to integrated service delivery (e.g., transitions of care, shared care planning, user experience with coordination) and is shared appropriately between relevant providers? | WI2, Assesses the availability and use of data specifically for monitoring integration. |
|  | IME3 | Are there established protocols and technological infrastructure (e.g., interoperable electronic health/social records, shared platforms) that facilitate the secure exchange of relevant client information between different health and social care providers involved in LTC? | WS3  Evaluates the operational capacity for information sharing to support integrated care. |
| 4. Workforce | WRK1 | Is there a national/regional workforce strategy for LTC that addresses the need for interdisciplinary skills, collaborative competencies, and new professional roles required for integrated care delivery? | SF, Evaluates strategic planning for a workforce capable of delivering integrated LTC. |
|  | WRK2 | Are there formal education and continuous training programs for health and social care professionals that include joint modules or specific content on integrated LTC, inter-professional collaboration, and person-centred care for complex needs? | SF, Assesses capacity building for integrated care within workforce development. |
|  | WRK3 | Are there clear definitions and supportive regulations for new or evolving professional roles (e.g., care coordinators/managers, advanced practice nurses in LTC, integrated care navigators) that facilitate integration across health and social sectors, and are the number of these practitioners monitored? | WI3, Evaluates the formalization of roles that bridge sectoral divides. |
| 5. Service Delivery | SRV1 | Are standardized person-centred assessment protocols to foster integrated care developed and being used? | WS1, Evaluates the use of comprehensive needs assessment protocol. |
|  | SRV2 | Do national or regional policies/guidelines define standardized processes and procedures for creating integrated and person-centred LTC pathways, including clear transition policies, case/care management protocols, and risk stratification for prioritization? | WS2, Evaluates the formalization of integrated care pathways at a systemic level. |
| 6. Innovation and Research | INR1 | Does national/regional policy foster the development and adoption of digital information technologies (e.g., telehealth, shared electronic records, mobile health applications) specifically to facilitate communication, information exchange, and coordination among sectors and stakeholders in LTC? | WI1, Evaluates policy support for technology as an enabler of integration. |
|  | INR2 | Is there a national/regional strategy or funding mechanism to promote research and innovation in integrated LTC models, including the evaluation of their effectiveness, cost-effectiveness, and scalability? | SF, Assesses commitment to evidence generation and learning in integrated LTC. |

## References

1. WHO. Framework for countries to achieve an integrated continuum of long-term care. 2021.

2. Leijten FR, Struckmann V, van Ginneken E, Czypionka T, Kraus M, Reiss M, et al. The SELFIE framework for integrated care for multi-morbidity: development and description. Health policy. 2018;122(1):12-22.

# Supplementary S2. Rationale and Process for Country and Area Selection

This supplementary note expands on the rationale and process by which the five countries—Australia, Japan, New Zealand, the Republic of Korea, and Singapore—were selected for comparative analysis. While the main text presents a concise summary, this supplement offers a more systematic and transparent account of the selection logic, including a stepwise narrowing approach based on exclusion criteria.

1. **Stepwise Countries and Areas Selection Logic: From Regional Scope to Final Cases**
   1. **Initial Scope – Western Pacific Region (WPR):**
      Based on the WHO classification, the study is regionally focused on the WHO Western Pacific Region (WPR), which includes 32 countries and territories. This scope provides geographic proximity, shared exposure to WHO guidance, and comparable regional development pressures.
   2. **Criterion 1: Presence of a Public Long-Term Care (LTC) Policy or Framework**
      Of the 32 WPR countries and areas, only 11 had reported (Australia, Cambodia, Cook Islands, Japan, Malaysia, New Zealand, Palau, Philippines, Republic of Korea, Singapore, and Viet Nam), by 2023, a national LTC policy, plan, strategy, or framework, either as a standalone document or as part of aging or health policies. These countries were identified using WHO’s global LTC monitoring database.

⮕ Countries and areas excluded at this stage include those without a formalized LTC policy and those that did not report or respond to WHO’s global LTC monitoring surveys^[[1]](#footnote-1)^.

- 1. **Criterion 2: Nationwide Public LTC Scheme Operational by 2020**
     To enhance comparability in terms of maturity of LTC schemes, five countries (Australia, Japan, New Zealand, Republic of Korea, and Singapore) who reported having a national LTC policy, plan, strategy, or framework, either as a standalone document or as part of aging or health policies were selected as final cases of comparison.

This further enhanced comparability in terms of socioeconomic context and LTC demand, as only high-income economies (World Bank classification) experiencing advanced population aging (≥14% of the population aged 65+) were included.

1. **Analytical Rationale for the Selected Group**

While these five countries are comparable in terms of regional location, economic development, and demographic trends, they represent divergent system archetypes in governance, financing, and integration with health systems. This constructive diversity supports the study’s objective: identifying system-level enablers and barriers for integrated care in varied contexts facing a common challenge.

The selected cases allow for horizontal comparisons (e.g., financing models, workforce integration) and vertical analyses of how LTC systems evolved in response to institutional path dependencies and policy priorities.

The five selected countries—Australia, Japan, New Zealand, the Republic of Korea, and Singapore—share key conditions that shaped their LTC reforms. All face advanced population aging and have experienced pressure from high inpatient service use, often due to “social admissions” linked to limited community-based care and generous hospital coverage. These pressures led to policy efforts to reduce bed-blocking and shift from facility-based models toward home- and community-based care (HCBC), promoting aging in place. These shared drivers offer a common foundation for comparing LTC system responses to integrated care challenges.

# Supplementary S3. Characteristics of the public long-term care system across five countries

**Supplementary Table ST3. Service delivery characteristics of the public long-term care system across five countries**

| Section | Topic | Australia | Japan | Korea | New Zealand | Singapore |
| --- | --- | --- | --- | --- | --- | --- |
| Settings for public LTC support |  | * While most people use HCBS (home and community-based services) (82.9%) ^1^, most money is spent on IC (institutional care) (57.5%) ^2^  * Community care (center-based services) is available ^1,3^  * Cash benefits are not available ^1^ | * Both HC (home care) and IC are available ^4^  * Community care (center-based services) is standard ^4^  * No cash benefits ^4^ | * Both HC and IC are available ^5^  * Community care (center-based services) is common  * Cash benefits are restricted ^5^ | * While most people use HC (71.4%), most money is spent on IC (64.9%)  * Community care (center-based services) is almost not available ^6,7^  * Cash benefits are not available ^8^ | * Both HC and IC are available ^9^  * Community care (center-based services)^9^  * Cash benefits are available ^9^ |
| Minimal services definition | * Common: HC – personal care (daycare, meals, bathing, nursing), IC - personal care + accommodations | | | | | |
| Integrated care and person-centered care pathways | Service Integration for person-centered care | * Several pilots are underway ^11^, but fragmentation of funding sources between HCBS and IC works as a barrier ^1,12^  * For entry-level HCBS services, the independent care assessor develops personalized care plan, and beneficiaries are free to choose providers ^13^  * For more intensive HCBS and IC, care plans are developed by service provider  ^14,15^ | * Community-based integrated care center and care managers develop personalized care plans, but beneficiaries are free to choose providers ^4,16^ | * Several pilots are underway to foster need-based service coordination ^17^  * NHIS (National Health Insurance Service) provides personalized standard service guidelines, but beneficiaries are free to choose providers ^18^ | * Some hospital services are integrated as LTC services (E.g., age-related/psychogeriatric hospitals) ^19^  * The government need assessor to develop a care plan service and a coordinator to identify the most appropriate services ^8^ | * First pilots of Single-point-of-contact (SPOC) to coordinate one care plan slated for 2025 with an intention to be rolled out nationally in 2026 ^20^  *Integrated Home and Day Care (IHDC) packages, which are expected to be broken down in 2026 supporting seniors with multiple care needs ^21^ |
| Quality assurance | * Assurance mechanism for each HC/IC provider  * Follow up or penalty | * Quality accreditation quality audits, site visits, and complaint investigations of IC/HCBS providers^22^  * Mandatory reporting of serious incidents by service providers ^22^  * Sanctions can be imposed for non-compliance ^1^ | * Minimum workforce and facility requirements to open IC/HC facilities ^4^  * Accidents should be reported to municipal governments ^4^ | * Minimum workforce and facility requirement to open IC/HC facilities ^18^  * NHIS operates a fee surcharge linked to quality assessment ^18^ | * Regulation based contract with gov’t to comply with the national standard for IC/HC ^23^  * No further regulations for HC, certification requirement for IC ^24^ | * No national regulations for HC and existing quality assurance legislations for IC ^25^ ^26^  * Audit mechanism embedded to oversee the service delivery ^25^ |

**Supplementary Table ST4. Workforce characteristics of the public long-term care system across five countries**

| Section | Topic | Australia | Japan | Korea | New Zealand | Singapore |
| --- | --- | --- | --- | --- | --- | --- |
| Existing Workforce | Distribution of PCA (personal care assistant), nurse, AHP, and administrative workers (%) | * 59, 13, 4, 25  in 2023 ^27,28^ | * 66, 17, 2, 11 in 2023 ^29^ | * 89, 4, 1, 7 in 2022 ^30^ | * 73, 18, 1 8 in 2023 ^31^ | * 69, 15, 15, NA in 2017 ^33^ |
|  | Age and gender of PCA | * Only 28% of them were aged over 50, and the majority (86%) of them were female in 2020 ^28^ | * The majority (53.2%) were aged between 40-59, and 73.9% of workers were female ^29^ | * The majority (74.2%) were aged between 50-69 and 94.4% of workers were female ^30^ | * The majority (HCBS (74.5%), IC (36.9%)) were aged between 45-64, and 96-97% of workers were female ^32^ | * The majority (40%) were aged between 40-59 years, and 73% of them were female ^33^ |
|  | Share of foreign workers | * 17% of nursing and personal care staff were temporary residents ^27^ | * Almost negligible, but increasing ^34^ | * Negligible | * 71% of IC had at least one migrant staff in 2023 ^31^ | *60% of overall formal care in 2010-2020) ^33^ |
| Capacity-building  and professionalization | Certification requirement | * No ^1^ | * Only required for physical care provision (i.e., touching the human body) ^29,35^ | * Yes, required to be PCA  (240 hours of training and exam) ^18^ | * Yes, employers need to ensure PCA can attain the qualification by law ^36^ | * Nurses are accredited as nurses, and social workers are certified as social care professionals, but not as LTC workers. ^33^  * Several institutions offering to train LTC social workers with accredited courses, which are also actively supported by the government. ^33^ |
|  | Career development of PCA | * No clear career pathways ^1^ | * The career grade system, which enables PCAs to develop their career as PCA skill assessors ^37^ | * Can be an administrator of HCBS organization if worked as a PCA for more than 5 years ^38^ | * PCA certificate level and payment increases as the working period is prolonged ^36^ | * No certificate required for nursing aide or healthcare assistant ^33^  *Skill-focused training available  ^33^ |
| Staff Turnover and Retention | Turnover rate (of PCA) | * 27% of nursing, personal care, and clinical care managers (IC/HCBS) in 2023 ^27^ | * 15% during 2012-2016 ^35^ | * Average tenure 3.3 years, part-time workers accounting for more than half in 2022 ^30^ | * 26% (IC, PCA) in 2021 ^39^  * HCBS: N/A | *Local workers: 3.4 years; foreign workers: 2.8 years),  *About 49% of local workers stay for under 2 years; About 55% of foreign workers stay for under 2 years; PCA working less than 5 years 90% ^33^ |
| Support for family carers |  | * Respite care, coaching, counselling, connecting with other carers, and online skills courses ^1^  * Carer cash payments ^1^  * National dementia referral and support service ^2^ | * Various programs supporting self-management and social connection ^40^ | * Respite care,  dementia carers targeted emotional and educational support program ^41^ | * Respite care ^8^ | * Respite care, training (subsidy provided), support groups ^42^ |

**Supplementary Table ST5. Financing characteristics of the public long-term care system across five countries**

| Section | Topic | Australia | Japan | Korea | New Zealand | Singapore |
| --- | --- | --- | --- | --- | --- | --- |
| Expenditure | Share of public IC and total LTC expenditure/GDP | * 0.9% and 1.2% in 2021 ^43^ | *1.3% and 2.0% in 2021 ^44^ | * 0.5% and 0.8% GDP in 2022 ^44^ | * N/A and 1.5% in 2021 ^45,46^ | * 0.1% and 0.19% of GDP in 2016 ^33^ |
| Population and Benefit Coverage | Population Coverage | * Universal who satisfy the need based on eligibility criteria, but age<65 is only accepted in exceptional cases (e.g., Indigenous Australians) ^1^ | * Universal who  satisfy need-based eligibility criteria, but age<65 is only accepted in exceptional cases (e.g., aged ≥40 having geriatric disease) ^4^ | * Universal who  satisfy need-based eligibility criteria, but age<65 is only accepted in exceptional cases ^5^ | * Universal who  satisfy need-based eligibility criteria, but age<65 is only accepted in exceptional cases (e.g., aged 50–64 single with no dependent children (IC)  ^8,47,48^) | * Universal Means-tested subsidies for Singaporean citizens who satisfy need-based eligibility criteria ^49^  ; lower subsidies for permanent residents ^49^ ^50^ |
|  | Benefit Coverage | * Gov’t covers 73.4% (IC)-95.3% (HCBS) of the expenditure in 2020/21 ^1,51^  * Means-tested co-payments with annual and lifetime caps ^2^ | * Copayments range from 10% (HCBS) to 30% (IC)  * Copayments are capped based on income, and HCBS benefits are also capped based on eligibility grades ^52,53^ | * Monthly cap of benefits based on eligibility grades for HCBS at 15% copayments, full coverage of IC at 20% copayments. Redemption of copayment based on income level ^18^ | * Gov’t covers 57% (IC) of the expenditure ^54^  * In general, center-based community care services are not available ^8,48^  a | * Gov’t coverage range 20-75% (IC), 30-80% (HCBS) based on income ^50^  * ElderShield covered about 1.3 million Singaporeans as of 2017, comprising 64% of the target population aged 40 to 84 ^49^ |
| Revenue raising | The primary source of funding | * General taxation from the federal government (79.2%) in 2020-21 ^51^ | * 50% of public funds are raised through taxation, and the other raised through LTCI ^53^ | * General taxation supports an additional amount equal to 20% of the total LTCI revenue  ^18^ | * General taxation 92%, OOP 8% (in 2009/2010) ^55^ | *Government spending 42% (means-tested government subsidies 26% + government subsidies to LTC providers 16%), OOP 40%, LTCI 9%, charitable donations 9% (2015, for elderly 65+) ^50^ |
| Pooling resources |  | * National level single pool ^1^  * Separated with healthcare funding ^56,57^ | * Municipality level multiple pool ^53^  * Separated with healthcare funding ^4^ | * National level single pool ^18^  * Separated with healthcare funding ^18^ | * National level single pool ^58^  * Integrated within healthcare funding ^58,59^ | * National level multiple schemes and subsidies exist ^60^  * Separated with healthcare funding ^50^, but health and care pooled at decentralized health cluster level |
| Purchasing goods and services |  | * National-level schedules of fees ^61^  * Complex formula for purchasing services (subsidy + basic fees + means test fees + extra purchase) ^1,56^ | * National-level fixed fee-schedule  * Marginal costs (facility type, number of users, hours of service used) are reflected in HCBS price ^52^  * Per diem for public IC ^52^  * Mechanisms to compensate losses (balance billing, region-based fee adjustment by conversion factor ^52^ | * National-level fixed fee-schedule  * Marginal rate per hour per day for HCBS and per diem for IC ^18^  * Balance billing is not allowed ^18^ | * IC: Nationally set basic-per diem which is different across regions. HC: upon contract (i.e., differ across regions) ^54^  * Per-diem for IC, (FFS or bulk-funded case-mix) + IBT (In-Between Travel) for HCBS ^54^ | * Market-priced fees ^62^  * Means-tested subsidy + government subsidy for the poor ^62^  * Decentralized health clusters have authorities to incorporate more flexible payment scheme such as bundled payments ^63^  * AIC (Agency of Integrated Care) on behalf of MoH (Ministry of Health) providing social care funding nationally ^64^ |

**Supplementary Table ST6. Governance characteristics of the public long-term care system across five countries**

| **Section** | **Topic** | **Australia** | **Japan** | **Korea** | **New Zealand** | **Singapore** |
| --- | --- | --- | --- | --- | --- | --- |
| Long-term care legislation and strategy | Legislation | * Aged Care (LTC) Act legislated and implemented since 1997 ^65^ | * The National LTCI Act was legislated 1997 and has been implemented since 2000 ^66^ | * National LTCI Act legislated and implemented since 2008 ^67^ | * The Residential Care and Disability Support Services Act 2018 has been legislated and implemented since 2018 ^68^ | * Maintenance of Parents Act in 1996 was considered as one of the first legislation for elderly care ^69^  * Mandatory LTCI act for cash-benefit legislated in 2019 ^49^ |
|  | National Strategy | * Exist and but not periodically updated ^70^ | * Exists and updated every 3 years ^4^ | * Exists and updated every 5 years ^67^ | * Exits, and frequently  but not periodically updated ^6^ | * Exits and annually updated ^62^ |
| are Governance structure | Governance structure | * Federal gov’t (DoHAC: Department of Health and Aged care) manages all as a single insurer ^1^ | * Federal gov’t (MHLW: Ministry of Health, Labour and Welfare) manages benefit packages, prices, eligibility, and service standards ^66^  * LGUs purchasing services as insurers ^4,66^ | * Federal gov’t (MoHW: Ministry of Health and Welfare) manages all as a single insurer and leads committee negotiating contribution rates, price level, and benefit packages ^18,67^ | * Federal gov’t (Health NZ: Health New Zealand) manages all as a single insurer  Following recent reform 2022 ^71^ | * MoH and MSF (Ministry of Social and Family Development) manages LTCI through Gov’t board ^50^  * AIC responsible for overall implementation and integration of initiatives ^64^  * Three regional health systems accountable for responsible population-based budget introduced in 2017 ^64^ |
| Accountability mechanisms | Accountability mechanisms for sustainable LTC structure | * Mandatory reporting of financial, workforce and operational information ^72^  * Financial and prudential monitoring, compliance, and intervention framework ^73^ | * LGUs face pressure since they bear the fiscal burden through local LTCI contribution and local taxation ^53^ | * MoEF (Ministry of Economy and Finance), provider association, contributor representatives attend the committee that decides on contribution rates ^67^ | * Without government compensation for deficits, providers impose extra or premium service charges ^54^ | * Expenditure report and projections for LTC expenditure are publicly available ^74^  * Competition mechanism among three healthcare clusters (patients free to choose institutions) ^75^ |

**S3 Reference**

1. Royal Commission, Australia. *Final Report: Care, Dignity and Respect - Volume 2 The current system*. 2021.

2. Department of Health and Aged Care, Australia. *2022–23 Report on the Operation of the Aged Care Act 1997*. 2023.

3. Department of Health and Aged Care, Australia. *Financial Report on the Australian Aged Care Sector 2022–23*. 2024.

4. Fu R, Iizuka T, Noguchi H. *Long-term Care in Japan*. NBER Working paper 31829. 2023.

5. National Health Insurance Service, Republic of Korea. *2023 Long-term care Insurance Statistical Yearbook 노인장기요양보험 통계연보*. 2024.

6. World Health Organization. *New Zealand: Health System Review*. 2022. 9290210125.

7. Health New Zealand. About Health New Zealand. Updated July 25, 2024. <https://www.tewhatuora.govt.nz/corporate-information/our-health-system/organisational-overview/about-health-new-zealand/>

8. Ministry of Health, New Zealand. *Needs Assessment and Support Services for Older People - What you need to know.* 2011.

9. Chan A. An Overview of Singapore’s Long-term Care System: Towards a Community Model Care. *Coping with Rapid Population Ageing in Asia*. ERIA; 2021. Accessed 31 Oct 2023. <https://www.eria.org/uploads/media/Books/2021-Coping-with-Rapid-Population-Ageing-Asia/9_Part1-Ch-5-Long-term-Care-Singapore.pdf>

10. Health New Zealand. Nationwide service specifications. Updated 31 July 2024. <https://www.tewhatuora.govt.nz/health-services-and-programmes/nationwide-service-framework-library/about-nationwide-service-specifications>

11. Productivity Commission, Australia. *Shifting the Dial: 5-year Productivity Review, Supporting Paper No. 5*. 2017.

12. Royal Commission, Australia. *Final Report: Care, Dignity and Respect - Volume 1 Summary and recommendations*. 2021.

13. myagedcare, Australia. What happens after assessment. September 12, 2024. https://www.myagedcare.gov.au/assessment/what-happens-after-assessment

14. Department of Health and Aged Care, Australia. Ongoing care discussions for residential aged care. Updated 22 January 2020. https://www.health.gov.au/our-work/residential-aged-care/managing-residential-aged-care-services/ongoing-care-discussions-for-residential-aged-care

15. Department of Health and Aged Care, Australia. Care plans for Home Care Packages. Updated 4 September 2024. <https://www.health.gov.au/our-work/hcp/care-management/care-plans#:~:text=This%20will%20help%20when%20choosing%20services>

16. Sano J, Hirazawa Y, Komamura K, Okamoto S. An overview of systems for providing integrated and comprehensive care for older people in Japan. *Archives of Public Health*. 2023;81(1):81.

17. Kim H, Kwon S. A decade of public long-term care insurance in South Korea: policy lessons for aging countries. *Health Policy*. 2021;125(1):22-26.

18. Kim H, Seo D, Yoon N, Yun S, Chun S, Seo E-S. *Reorganization of the care system for the older aged population in the super-aged society: A study on reorganization to an integrated health and care system. 초고령사회 노인 돌봄체계 개편 방향: 의료-돌봄 통합체계로의 개편 방안 연구*. 2021.

19. eldernet. Where from here - Essential information for older people, Upper North Island. 2023.

20. Chew N. The Three Zones. 2019 [cited 1 Aug 2024]. In: River of Life [Internet]. Singapore: NHG, [cited 1 Aug 2024]. Available from: <https://corp.nhg.com.sg/ROL/Documents/ROL_Chapter%203.pdf>.

21. Agency for Integrated Care, Singapore. Who Is AIC. 2023. Available from: <https://www.aic.sg/about-us>

22. Aged Care Quality and Safety Commission, Australia. *Sector performance report Quarter 4 , April–June 2024*. 2024.

23. Ministry of Health, New Zealand. Resources for Ngā Paerewa Health and Disability Services Standard. Updated 10 March 2023. <https://www.health.govt.nz/regulation-legislation/certification-of-health-care-services/standards/nga-paerewa-health-and-disability-services-standard>

24. Meehan B, Millar N. Regulating the quality of long-term aged care in New Zealand. *Regulating Long-Term Care Quality: An International Comparison*. 2014;

25. Ministry of Health, Singapore. DIRECTIVES FOR QUALITY ASSURANCE COMMITTEES FOR NURSING HOMES: REGULATION 12A(1)(B) OF THE PRIVATE HOSPITALS AND MEDICAL CLINICS REGULATIONS (CAP 248, RG 1), 2011. Available at: <https://www.moh.gov.sg/licensing-and-regulation/regulations-guidelines-and-circulars/details/directives-for-quality-assurance-committees-for-nursing-homes-regulation-12a(1)(b)-of-the-private-hospitals-and-medical-clinics-regulations-(cap-248-rg-1)-dated-02-jan-2011>

26. Chong NW. *COMMUNITY-BASED LONG TERM CARE IN SINGAPORE*. Accessed 31 Oct 2023. <https://www.tma.tw/homeMedical/files/%E6%96%B0%E5%8A%A0%E5%9D%A1-Community-based%20Long%20Term%20Care%20in%20Singapore%20Nov%2017-%E7%A0%94%E8%A8%8E%E6%9C%83%E5%A0%B1%E5%91%8A%E7%89%88.pdf>

27. Australian Institue of Health and Welfare. *2023 Aged Care Provider Workforce Survey Summary report*. 2024.

28. Department of Health, Australia. *Aged care workforce census report*. 2020.

29. Long-term care Labor Stabilization Center, Japan. Survey on the Employment Status and Attitudes of Long-term Care Workers 介護労働者の就業実態と就業意識調査 2024 [Available from: <https://www.kaigo-center.or.jp/content/files/report/2023_jittai_chousa_roudousya_honpen.pdf>]

30. Lee YK, Lee SH, Kang E, Kim SJ, Namkung EH, Choi Y. *2022 Long-Term Care Survey. 2022년 장기요양 실태조사*. 2022. <https://www.mohw.go.kr/board.es?mid=a10411010100&bid=0019&act=view&list_no=378319&tag=&nPage=1>

31. New Zealand Aged Care Association. *Aged residential care sector profile 2024*. 2024.

32. The New Zealand Work Research Institute. *The New Zealand Aged Care Workforce Survey 2016*. 2016.

33. Lien Foundation, Singapore. *Long Term Care Manpower Study*. 2018. July 2018. Accessed 31 Oct 2023. <https://www.lienfoundation.org/sites/default/files/Long%20Term%20Care%20Manpower%20Study%20FINAL_0.pdf>

34. Operating Committee of the Council for Specified Skilled Workers in the Long-Term Care Sector, Japan. Records of Foreign Worker Acceptance in the Long-Term Care Sector 介護分野における 外国人の受入実績等 2022 [Available from: <https://www.mhlw.go.jp/content/12000000/001090473.pdf>]

35. Takeda S. Factors related to turnover and intention to leave the care working profession in Japan: a review. *Yonago Acta Medica*. 2023;66(2):196-201.

36. Careerforce. FAQs>What qualification do you need to work in Home and Community Support Work? July 31, 2024. <https://www.careerforce.org.nz/our_sectors/home-community-support/>

37. Elderly Service Providers Association, Japan. Long-term Care Professional Career Grade System 介護プロフェッショナルキャリア段位制度. Tokyo: 2023 [Available from: <https://careprofessional.org/careproweb/summary>

38. Ministry of Health and Welfare, Republic of Korea. Enforcement rules of Welfare of Senior citizens act. 노인복지법 시행규칙. Accessed July 29, 2024, <https://www.law.go.kr/lsSc.do?section=&menuId=1&subMenuId=15&tabMenuId=81&eventGubun=060101&query=%EB%85%B8%EC%9D%B8%EB%B3%B5%EC%A7%80%EB%B2%95#J16316649>

39. New Zealand Aged Care Association. *Aged Residential Care Industry Profile 2020–2022*. 2022.

40. Japan International Cooperation Agency. Community-based Integrated Care in Japan. In: Hou X, Sharma J, Zhao F, eds. *Silver Opportunity: Building Integrated Services for Older Adults around Primary Health Care*. World Bank Publications; 2023.

41. Han E-J, Park M, Park S, Han D. Randomized controlled trial of the caregiver orientation for mobilizing personal assets and strengths for self-care (COMPASS) for caregiving journey: a national family caregiver support program in a long-term care insurance system. *Journal of the American Medical Directors Association*. 2020;21(12):1906-1913. e3.

42. Agency for Integrated Care, Singapore. Day care. 2023. Available from: <https://www.aic.sg/care-services/day-care>

43. OECD Statistics. Health expenditure and financing. 23 July, 2024. Accessed 23 July, 2024. <https://stats.oecd.org/Index.aspx?DataSetCode=SHA>

44. OECD. OECD Data Explorer. July 31, 2024. 2024. <https://data-explorer.oecd.org/>

45. OECD. *Health at a Glance 2021: OECD Indicators*. 2021. https://doi.org/10.1787/ae3016b9-en

46. OECD. *Health at a Glance 2013: OECD Indicators*. 2013. https://doi.org/10.1787/ae3016b9-en

47. New Zealand Government. Help in your home / Cooking and meals. 25 July, 2024. <https://www.govt.nz/browse/health/help-in-your-home/cooking-and-meals/>

48. Ministry of Health, New Zealand. *Long-term residential care for older people: What you need to know*. 2019.

49. Fong JH, Borowski A. Long-Term Care Insurance Reform in Singapore. *Journal of Aging & Social Policy*. 2022/01/02 2022;34(1):73-90. doi:10.1080/08959420.2021.1926867

50. Graham WCK, Bilger M. Financing Long-Term Services and Supports: Ideas From Singapore. *Milbank Q*. Jun 2017;95(2):358-407. doi:10.1111/1468-0009.12264

51. Department of Health and Aged Care, Australia. *Financial Report on the Australian Aged Care Sector 2020–21*. 2022.

52. Ikegami N. Case study: Japan. In: Barber S, ed. *Pricing long-term care for older persons*. World Health Organization; 2021.

53. Ministry of Health LaW, Japan. Long-term care insurance system of Japan. Accessed July 25, 2024, <https://www.mhlw.go.jp/english/policy/care-welfare/care-welfare-elderly/dl/ltcisj_e.pdf>

54. Moore D, Loan J, Rohani M, Trill R, Manning N, Yee D. A review of aged care funding and service models. 2024. <https://www.tewhatuora.govt.nz/for-health-professionals/clinical-guidance/specific-life-stage-health-information/health-of-older-people/aged-care-funding-and-service-models-review/#downloads>

55. New Zealand Treasury. *Long-Term Care and Fiscal Sustainability*. 2013.

56. Barber SL, van Gool K, Wise S, et al. *Pricing long-term care for older persons*. World Health Organization; 2021.

57. Department of Health and Aged Care, Australia. Budget 2023–⁠24: Building a stronger Medicare. <https://www.health.gov.au/ministers/the-hon-mark-butler-mp/media/budget-2023-24-building-a-stronger-medicare>

58. Ministry of Health, New Zealand. Vote Health. Updated May 30, 2024. <https://www.health.govt.nz/about-ministry/what-we-do/vote-health>

59. Health New Zealand. Aged Care Funding and Service Models Review. Updated April 23, 2024. <https://www.tewhatuora.govt.nz/for-health-professionals/clinical-guidance/specific-life-stage-health-information/health-of-older-people/aged-care-funding-and-service-models-review/>

60. Ministry of Health, Singapore. HEALTHCARE SCHEMES & SUBSIDIES (2023). Available at: <https://www.moh.gov.sg/healthcare-schemes-subsidies>

61. Department of Health and Aged Care, Australia. Schedule of Fees and Charges for Residential and Home Care. Updated June 28, 2024. <https://www.health.gov.au/resources/publications/schedule-of-fees-and-charges-for-residential-and-home-care?language=en>

62. Graham WCK, Bilger M. Financing Long-Term Services and Supports: Ideas From Singapore. *The Milbank Quarterly*. 2017;95(2):358-407. doi:https://doi.org/10.1111/1468-0009.12264

63. Wyse M. Financing Long-Term Care in Asia and the Pacific. Asian Development Bank Brief; 2021.

64. Asian Development Bank. Singapore's long-term care system adapting to population aging. 2020.

65. Department of Health and Aged Care, Australia. Aged care laws in Australia. Department of Health and Aged Care,. Updated 30 November 2023. <https://www.health.gov.au/topics/aged-care/about-aged-care/aged-care-laws-in-australia#other-aged-care-laws>

66. Ministry of Justice, Japan. Long-Term Care Insurance Act. Accessed July 25, 2024, <https://www.japaneselawtranslation.go.jp/en/laws/view/3807/en>

67. Ministry of Health and Welfare, Republic of Korea. Long-term Care Insurance Act. 노인장기요양보험법. . Accessed July 29, 2024, <https://www.law.go.kr/LSW/lsSc.do?section=&menuId=1&subMenuId=15&tabMenuId=81&eventGubun=060101&query=%EB%85%B8%EC%9D%B8%EC%9E%A5%EA%B8%B0%EC%9A%94%EC%96%91%EB%B3%B4%ED%97%98%EB%B2%95#undefined>

68. Holmes D. Spider Web and I'm Caught in the Middle: Older Persons, Residential Care, and the Family Trust-A New Zealand Perspective. *J Aging L & Pol'y*. 2021;12:83.

69. Hsu L. The Law and the Elderly in Singapore: The Law on Income and Maintenance for the Elderly. *Singapore Journal of Legal Studies*. 2003;(2)

70. Department of Health and Aged Care, Australia. Aged care reforms. Updated November 2022. https://oia.pmc.gov.au/published-impact-analyses-and-reports/aged-care-reforms

71. Health New Zealand. About the health reforms. Updated June 30, 2024. <https://www.tewhatuora.govt.nz/corporate-information/our-health-system/organisational-overview/about-the-health-reforms/>

72. Department of Health and Aged Care, Australia.. Aged care provider reporting. Updated 27 August 2024. <https://www.health.gov.au/topics/aged-care/providing-aged-care-services/reporting>

73. Department of Health and Aged Care, Australia.. Financial and Prudential Monitoring, Compliance and Intervention Framework. Updated 11 July 2023. <https://www.health.gov.au/our-work/aged-care-financial-viability-and-capability/financial-and-prudential-monitoring-compliance-and-intervention-framework#:~:text=The%20Financial%20and%20Prudential%20Monitoring,%20Compliance>

74. Ministry of Health, Singapore. GOVERNMENT'S OPERATING EXPENDITURE FOR LONG-TERM CARE SERVICES. 2020. Available from : <https://www.moh.gov.sg/news-highlights/details/government's-operating-expenditure-for-long-term-care-services>

75. Ministry of Health, Singapore. CAPITATION. 2023. Available from: <https://www.moh.gov.sg/news-highlights/details/capitation>

1. [Percentage of countries that have a long-term care policy / plan / strategy / framework (stand-alone or integrated within an ageing and health plan)](https://platform.who.int/data/maternal-newborn-child-adolescent-ageing/indicator-explorer-new/mca/percentage-of-countries-that-have-a-long-term-care-policy-plan-strategy-framework-(stand-alone-or-integrated-within-an-ageing-and-health-plan)) [↑](#footnote-ref-1)
